# Grouping of UVCB Substances with Dose-Response Transcriptomics Data from Human Cell-Based Assays

**DOI:** 10.14573/altex.2107051

**Published:** 2022-03-10

**Authors:** John S. House, Fabian A. Grimm, William D. Klaren, Abigail Dalzell, Srikeerthana Kuchi, Shu-Dong Zhang, Klaus Lenz, Peter J. Boogaard, Hans B. Ketelslegers, Timothy W. Gant, Ivan Rusyn, Fred A. Wright

**Affiliations:** 1 Bioinformatics Research Center, North Carolina State University, Raleigh, NC, USA; 2 Department of Veterinary Integrative Biosciences, Texas A&M University, College Station, TX, USA; 3 Public Health England, Centre for Radiation, Chemical and Environmental Hazards, Harwell Science Campus, Oxon, UK; 4 Northern Ireland Centre for Stratified Medicine, Ulster University, L/Derry, Northern Ireland, UK; 5 SYNCOM Forschungs und Entwicklungsberatung GmbH, Ganderkesee, Germany; 6 SHELL International BV, The Hague, The Netherlands; 7 Concawe, Brussels, Belgium; 8 current address: Biostatistics & Computational Biology Branch, National Institute of Environmental Health Sciences, RTP, NC, USA; 9 current address: ToxStrategies, Inc., Asheville, NC, USA; 10 current address: MRC-University of Glasgow Centre for Virus Research, Glasgow, Scotland, UK

## Abstract

The application of *in vitro* biological assays as new approach methodologies (NAMs) to support grouping of UVCB (unknown or variable composition, complex reaction products, and biological materials) substances has recently been demonstrated. In addition to cell-based phenotyping as NAMs, *in vitro* transcriptomic profiling is used to gain deeper mechanistic understanding of biological responses to chemicals and to support grouping and read-across. However, the value of gene expression profiling for characterizing complex substances like UVCBs has not been explored. Using 141 petroleum substance extracts, we performed dose-response transcriptomic profiling in human induced pluripotent stem cell (iPSC)-derived hepatocytes, cardiomyocytes, neurons, and endothelial cells, as well as cell lines MCF7 and A375. The goal was to determine whether transcriptomic data can be used to group these UVCBs and to further characterize the molecular basis for *in vitro* biological responses. We found distinct transcriptional responses for petroleum substances by manufacturing class. Pathway enrichment informed interpretation of effects of substances and UVCB petroleum-class. Transcriptional activity was strongly correlated with concentration of polycyclic aromatic compounds (PAC), especially in iPSC-derived hepatocytes. Supervised analysis using transcriptomics, alone or in combination with bioactivity data collected on these same substances/cells, suggest that transcriptomics data provide useful mechanistic information, but only modest additional value for grouping. Overall, these results further demonstrate the value of NAMs for grouping of UVCBs, identify informative cell lines, and provide data that could be used for justifying selection of substances for further testing that may be required for registration.

## 1 Introduction

Substances classified as UVCBs (unknown or variable composition, complex reaction products, and biological materials) comprise over 20% of chemical registrations in Europe and present difficult challenges for hazard and risk evaluations (ECHA, 2017). Petroleum substances are UVCBs with a complexity that arises primarily from the presence of very large – hundreds to as many as millions – numbers of isomeric chemical constituents. The physicochemical processes during oil refining are complex, and varying sources of crude oil are used at different times in manufacturing facilities. For this reason, petroleum substance groupings and CAS numbers are typically based on physicochemical properties and performance characteristics rather than chemical characterization of the constituents (Salvito et al., 2020), albeit petroleum substances are made up of a few classes of hydrocarbons (alkanes, iso-alkanes, cyclo-alkanes and (poly)aromatics), and the actual chemical variation is highly determined by the physicochemical properties. Current practice to harmonize the identification of potential hazards among petroleum UVCBs is based on broad product categories (CONCAWE, 2020) that are largely informed by the product performance criteria, manufacturing processes, and the presence of polycyclic aromatic compounds (PAC) and other potentially hazardous constituents (Clark et al., 2013; McKee et al., 2015). However, regulatory agencies question the application of groupings and read-across for UVCB product categories due to insufficient justification for considerations of chemical or biological sameness of the products in each category (ECHA, 2020). Therefore, alternative data streams have been proposed as potentially relevant for supporting grouping of petroleum UVCBs to improve chemical (Grimm et al., 2017; Roman-Hubers et al., 2021; Onel et al., 2019) and hazard characterization (House et al., 2021), and to ultimately reduce and refine the need for new animal testing for registration of these products.

Several previous studies tested the hypothesis that grouping of complex substances and environmental mixtures can be achieved using data from *in vitro* assays in induced pluripotent stem cell (iPSC)-derived and other cell types (Grimm et al., 2016, 2019; Chen et al., 2020). This previous work demonstrated that despite the inherent complexity and the variability of samples, *in vitro* data can be used to discern informative biological patterns corresponding to chemical composition or manufacturing categories. In addition, these studies suggested that the diversity and physiological relevance of the data from studies in only a few iPSC-derived cell types such as hepatocytes (Grimm et al., 2015) and cardiomyocytes (Burnett et al., 2019) can yield sensitive multi-dimensional information to aid in grouping, providing a strong rationale and basis for future read-across efforts and prioritization of substances within manufacturing categories.

More recently, House et al. (2021) combined all these aspects in a comprehensive effort to investigate 141 substances, a compendium of samples comprising the majority of petroleum-based UVCBs registered under the Regulation on Registration, Evaluation and Authorisation of Chemicals (REACH) in the European Union (CONCAWE, 2019). That study generated *in vitro* bioactivity data from 15 human cell types as new approach methodologies (NAM) data to support substance grouping into 16 major categories of petroleum-based UVCBs. Extensive quality control was used to determine assays, including those specific to cell type, that were most informative and provided discernible dose-response relationships. The outcomes of this study showed that overall summaries of bioactivity yielded substance rankings concordant with their chemical composition and expected hazard potential as obtained from physical and analytical chemistry data. Moreover, unsupervised and supervised analyses suggested that the bioassay data provided important additional information relevant to the substance categorization; bioassay data alone appeared as informative to this categorization as traditional physicochemical data.

These observations were critical in identifying the most informative cell types and bioassays, providing potential cost savings in future studies. It is important to recognize that the ability of *in vitro* bioassays to provide relevant information may have only a partial relationship to *in vivo* relevance and health risk (ECHA, 2020). For example, data from iPSC-derived cardiomyocytes were among the most sensitive with respect to their ability to provide concordance with a manufacturing category (House et al., 2021). When aggregating across several *in vitro* data types, iPSC-derived hepatocytes showed a strong concordance between overall activity and PAC content, a known *in vivo* indicator of potential human health hazard (McKee et al., 2015). These observations further support the use of *in vitro* bioactivity assays as providing a potential framework for prioritization of substances within manufacturing categories but also highlight the need for additional mechanistic, such as gene expression, evidence of the effects to enable *in vivo* translation.

Overall, the previous results have strongly supported the utility of *in vitro* NAM for interrogating and grouping complex substances, including petroleum-based UVCBs. The eventual purpose of these groupings is to support read-across, and mechanistic evidence for the nature of perturbations underlying the bioassay phenotype responses to chemicals is gaining prominence in decision-making (Samet et al., 2020). In this context, we expect these data to inform read-across hypotheses and prioritize substances for *in vivo* testing. A refined understanding of the chemical effects at the intracellular level could potentially provide more informative *in vitro* models to characterize UVCBs, reducing the number of cell types and assays even further while retaining the ability to prioritize UVCBs for further testing within categories and to serve as a basis for read-across. The use of *in vitro* transcriptomic profiling of UVCBs is a natural step to provide this biological context. Indeed, the use of transcriptomics in toxicology is now well-established (Joseph, 2017), and this data stream is an increasingly popular NAM (Harrill et al., 2019). High-throughput transcriptomics has been used to interrogate biological effects of a large number of chemicals and perform transcriptomics dose-response analyses (Harrill et al., 2021; House et al., 2017). Previous high-throughput transcriptomic data of some petroleum-derived UVCBs showed that *in vitro* gene expression changes were specific to broad categories (e.g., heavy fuel oils vs. straight run gas oils) (Grimm et al., 2016).

In this study, we tested the hypothesis that transcriptomic profiles can be used to support grouping of petroleum substances and provide informative mechanistic data for existing groupings based on manufacturing class. For this, 141 petroleum substances, previously analyzed using extensive bioassays across a multitude of human cell types, were used as representative UVCBs. These substances were interrogated using transcriptomic profiling in six cell types in a dose-response design.

## 2 Materials and methods

### Chemicals

All chemicals used in these studies, except for petroleum substances, were obtained from Sigma-Aldrich (St. Louis, MO), unless otherwise noted. Samples of petroleum substances were supplied by Concawe (Brussels, Belgium). To enable *in vitro* studies of petroleum substances, extraction of petroleum substances into dimethyl sulfoxide (DMSO) was performed using American Society for Testing and Materials standard procedures (ASTM International, 2014). The DMSO extraction used herein was designed to concentrate the “biologically active” fraction (i.e., mostly 3–7 ring PAC, but also other polar constituents) of each petroleum substance; the extracts obtained using this method are used routinely for safety testing (e.g., mutagenicity) and chemical characterization of the refinery streams (CONCAWE, 1994). Briefly, 4 g of each tested petroleum substance (Tab. 1) was first dissolved in 10 mL of cyclohexane; 10 mL of DMSO (Fisher Scientific, Waltham, MA) was added, and the mixture was vigorously shaken for several minutes. The DMSO layer was removed using a glass pipette, and the cyclohexane was re-extracted with an additional 10 mL of DMSO. Both PAC-enriched DMSO layers were combined and diluted 2:1 with two volumes of 4% (w/v) sodium chloride solution. Following subsequent extraction with 20 mL and 10 mL cyclohexane to isolate the PAC fraction, the organic layers were washed twice with distilled water and filtered through anhydrous sodium sulfate. The procedure of extraction was also performed without addition of the petroleum substances, and the resulting fraction was designated as “vehicle” (method blank) to be used as a reference for comparisons. Petroleum substance extracts were further diluted to enable concentration-response testing.

### Study design

Overall, this study conducted concentration-response over 4 points (3 serial 1-log_10_ dilutions of each extract performed in duplicate, and ~45 vehicle controls for each extract). All samples were aliquoted into 384-well “master” plates (Masterblock 384-well, V bottom, Deepwell polypropylene plate; Cat. No. 781271; Greiner Bio-One North America, Monroe, NC) as detailed elsewhere (House et al., 2021). Plates were sealed with aluminum film and stored at −80°C until use. Copies of each master plate were prepared for use in all *in vitro* experiments. The final concentration of DMSO in assay wells following addition of test substances was 0.25–0.5% (v/v), depending on the cell type, see House et al. (2021) for details.

### Cell types

A total of 6 human cell types were used in these experiments (Tab. S1^1^). Cell type and vendor selections were based on the following considerations. Cells were chosen to be of human origin and to represent diverse organs/tissues. We used both iPSC-derived cells as well as established cell lines. These *in vitro* models had to be reproducible (i.e., a particular cell/donor can be obtained from a commercial source) and suitable for evaluation of both “functional” and “cytotoxicity” endpoints so that the specificity of the effects of test compounds could be assessed. Four of these cell types (hepatocytes, endothelial cells, neurons, and cardiomyocytes) were human iPSC-derived (FujiFilm-CDI, Madison, WI). Two cell types (A375 malignant melanoma cells and MCF7 breast cancer cells) were from ATCC (Manassas, VA). All cells were cultured as detailed elsewhere (House et al., 2021), and additional cell culturing information is given in the supplementary information^1^. Cells were plated in 384-well plates in densities recommended by the supplier, using optimized media supplied by the same company or optimized for density by experimentation for each cell line. Cells were cultured without treatment for a period of time required to achieve functional capacity. Plating density, cell culture conditions and duration are detailed elsewhere (House et al., 2021). Cells were treated with test substances in a series of dilutions to evaluate concentration-response as described above.

### Transcriptomics – Quality control

**Fig. 1:**
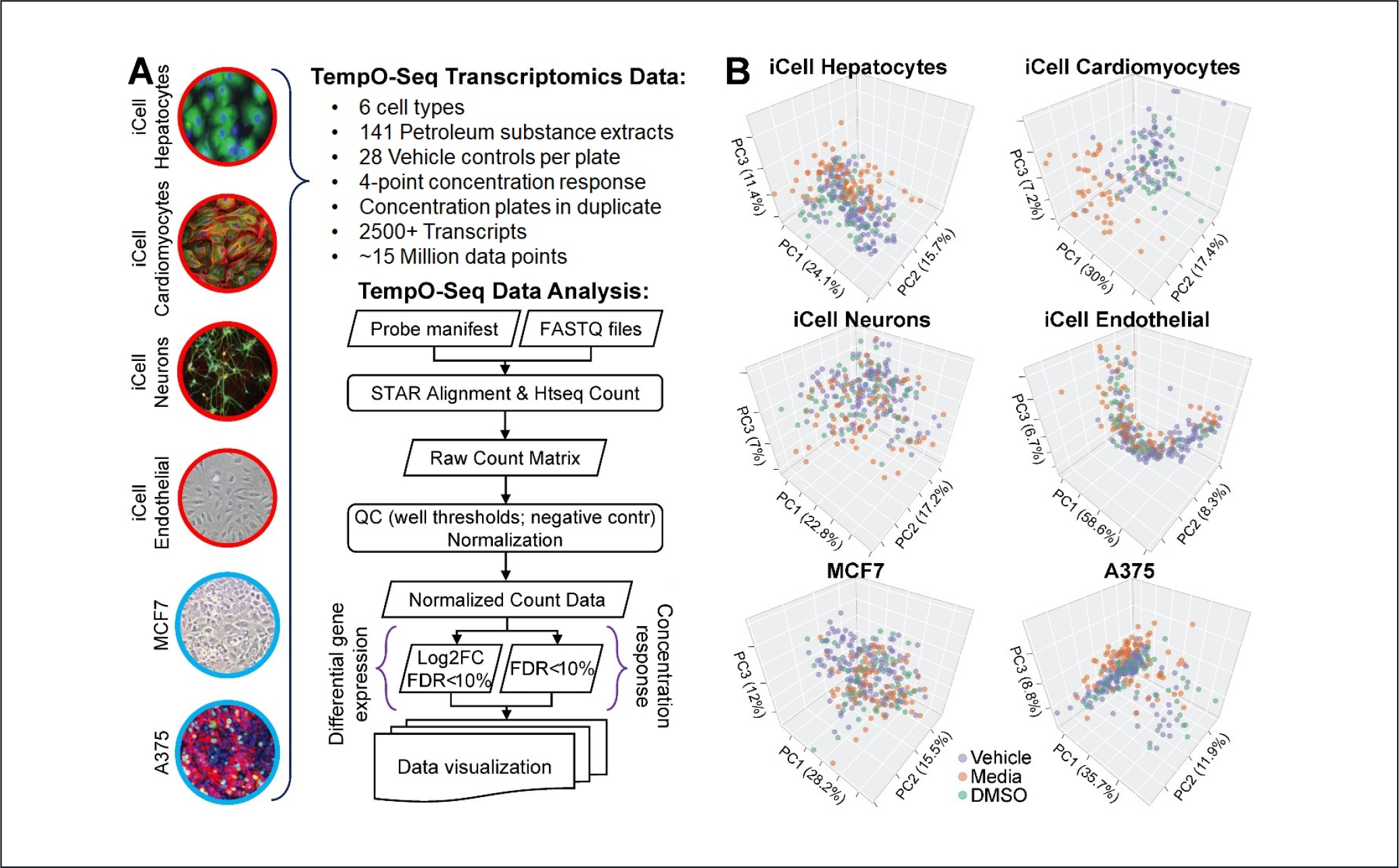
Overview of the multi-cell *in vitro* transcriptomic analysis of the effects of 141 petroleum substances (A) Summary of the dataset and data processing pipeline. (B) Principal component analysis of negative control (vehicle (method blank), media, or DMSO, see Section 2 for description) gene expression signatures in each cell type.

Overall plate design was explained previously in detail (House et al., 2021). In brief, the transcriptomic experiments were treated similarly but with only 6 cell types (iCell hepatocytes, iCell cardiomyocytes, iCell neurons, iCell endothelial, MCF7, and A375) and without the highest dose (undiluted extracted UVCBs) that elicited cellular toxicity for some cell type/treatment combinations in phenotypic assays. Raw sequenced reads were aligned and counted using the pipeline developed by our group (House et al., 2017). Samples with < 100K counts were removed, as were probes not expressed across at least 5% of the sample space. For the few genes with more than 1 probe, counts were summed to the gene level. At the gene level, these steps resulted in ~2,500 genes per cell type assessed for differential gene expression (DEG) and concentration response (CRG) for each of 141 UVCBs. Each plate contained three types of controls: media alone – “media”, media with DMSO – “DMSO”, and method blanks – “vehicle”. See House et al. (2021) for more detail. Within a cell type, these three types of controls were examined for correlation between each other (across the transcriptomic space), and controls whose correlation exceeded 3 standard deviations from mean correlation were removed as outliers. All three control types were examined with principal component analysis (PCA) of the top 500 expressed genes. PCA scatter plots (Fig. 1) revealed little difference between vehicle and DMSO, and thus vehicle controls were used as the most appropriate reference for all subsequent data analyses. For this publication, after quality control (QC), vehicle controls included 40–48 replicates (23–24 for iCell cardiomyocytes), providing a solid anchor for dose-response quantification followed by treatment dose response replication of the remaining serial dilutions. We highlight that this approach, applied over multiple doses, six cell lines, and ~2,500 genes provides considerable resolution for analyses of substances used in this study. In this study, the primary unit of observation was not a gene, but a substance. Each substance was interrogated over multiple doses and cell types, with nearly 90,000 expression data points, providing substantial replication for the observations of interest.

### Transcriptomics – Differential gene expression

For each cell type, the complete normalized count matrix of 141 UVCBs and vehicle controls was calculated using DESeq2 (Love et al., 2014). The maximum dose was compared to vehicle controls for each cell type/treatment/gene combination, and log2-fold-change values and p-values were calculated with an equal variance assumption. False discovery q-values were calculated from the p-values of each vector using the R v4.1 p.adjust function (method=”BH”) of roughly 2,500 genes for a given UVCB treatment/cell type combination; and FDR ≤ 0.1 was considered as a cut-off to identify expression-responsive gene(s) for a given cell type/UVCB combination. Normalized counts from DESeq2 were subsequently used for examination of dose-response criteria.

### Transcriptomics – Concentration response

We applied a set of statistical flags described in detail in House et al. (2017) to assess whether a concentration-response calculation was warranted for each cell type/gene/treatment combination. Linear modeling using DESeq2 (Love et al., 2014) was then conducted of log_2_(count + 0.5) transformed counts against log_10_(concentration) for each retained cell type/treatment/gene. In all cases, the control concentration was converted to the lowest treatment concentration divided by 10. For example, if the three concentrations given were 1/10 stock, 1/100 stock, and 1/1,000 stock, the vehicle control concentration was considered as 1/10,000, and concentration was coded as a predictor vector of −4, −3, −2, and −1 after log_10_ transformation. For a given treatment and cell type, false discovery q-values for linear trend (positive or negative) for concentration were calculated across all assessed genes as described above for a given cell type/treatment, and genes with FDR ≤ 0.1 were considered as concentration responsive.

### Pathway analysis

Pathway analysis and visualization was conducted in R with the xgr package (Fang et al., 2016) version 1.1.8 using Reactome Ontologies as gene sets. For both differentially expressed genes (DEGs) and concentration responsive genes (CRGs), an FDR of 5% was used to conduct pathway analysis. The gene set background was considered to consist of all TempOSeq-interrogated genes retained after low-count removal for a given cell type. Subsequently, pathway enrichment was conducted against the C2Reactome ontology sets.

### Polycyclic aromatic compound (PAC) analysis

Weight percentages of PAC in all tested samples were determined by gas chromatography-coupled mass selective detection (GC/MSD) as detailed previously (Roy et al., 1988). Briefly, each substance was extracted as detailed above and dried. The amount of each extract was then determined using the weight difference of the empty flask and following solvent evaporation. The extract was then dissolved in cyclohexane to a final concentration of 50 mg/mL and used for analytical assays. Sample separation was achieved on a Zebron-5HT capillary column (30 m; 0.25 mm; 0.25 mm; Phenomenex, Torrance, CA). Quantitative integration of the chromatograms was achieved using standards of naphthalene, phenanthrene, 1,2-benzanthracene, benzo[a]pyrene, bebenzo[g,h,i]perylene, and coronene. The resulting PAC profiles consist of weight percentages by ring number and are listed elsewhere (House et al., 2021).

### Establishing correspondence to bioactivity

The focus of this study was to characterize changes in gene expression in response to treatment, and comparison across UVCB categories, and not the bioactivity measures that were the subject of the earlier report (House et al., 2021). However, to establish the relevance of the results with the current design and the effect of sampling variation, we computed overall summed bioactivity across the 42 assays from the 15 cell types reported earlier (House et al., 2021). Summed bioactivity was the summed values over the 42 assays, where each bioactivity phenotype was standardized to a common unit variance, and large values correspond to high activity throughout. As the unit of study is each of the 141 UVCBs, we ran a cross-validated regression tree model for predicting bioactivity using gene responsiveness to treatment as a predictor for all ~2,500 TempOSeq genes, where the model was trained within each of the cell types. For regression trees, we used the *xgboost* R package (v 04.4) with default settings, and leave-one-out cross-validation to obtain predictions without overfitting. Prediction accuracy was recorded as the Pearson correlation r between true and predicted summed bioactivity. We interpret high correlations between predicted and actual bioactivity as indicative of high experimental reproducibility in this NAM system, even when the ground truth of biological effects is unknown.

### Supervised category analysis

As detailed in House et al. (2021), we trained a machine-learning statistical model to predict the existing categories (CONCAWE, 2019, 2020) of petroleum-based UVCBs under REACH (Tab. 1). As iCell hepatocytes appeared to be the most sensitive cell type in overall gene expression changes in this experiment, we performed analysis using the Prediction Analysis of Microarrays (PAM) package in Rv3.6 (Tibshirani et al., 2002) using iCell hepatocyte expression data for the 141 UVCBs, along with the 8 PAC and 42 bioassay phenotypic measurements from House et al. (2021), using (a) expression data alone, (b) expression + PAC, (c) expression + bioassay measurements, and (d) expression + PAC + bioassay measurements. Leave-one-out cross-validation was performed due to the small minimum category sizes in some instances, and we used a PAM shrinkage threshold of z = 1.28 for construction of the classifier. For each instance, we computed two measures of classification accuracy: the exact matching accuracy (proportion of matches of cross-validated category assignment vs. true assignment) from the associated confusion matrix, and the proportion correctly assigned to one of two major hazard groups. For the latter, we used an ordering of the categories in comparison to PAC to establish a group cutpoint (between bitumens and base oils), as shown below in Section 3. For the exact match criterion, the 95^th^ percentile of null permutations was computed in House et al. (2021) as 0.163 and used for statistical significance testing.

## 3 Results

Previous studies of petroleum-derived UVCBs have shown that combinations of analytical data (e.g., PAC content) and cell-based bioassays can be used to group, categorize, and largely recapitulate manufacturing-based classifications (House et al., 2021; Grimm et al., 2016). This study further extends knowledge on the application of *in vitro* data for grouping of complex substances by including transcriptional response measures for a variety of cell types in a dose-escalation design. While it is well established that transcriptomics provides direct insight into underlying mechanisms of response (Harrill et al., 2021; Yauk et al., 2020), the sensitivity and specificity of using expression profiles for grouping of complex UVCBs, in comparison with previous bioactivity measures, has not been explored.

The overall schematic of the study design and data processing pipeline are shown in Figure 1. Six cell types (iCell hepatocytes, iCell cardiomyocytes, iCell neurons, iCell endothelial, MCF7, and A375) were subjected to the 4-point treatment (3 dilution concentrations and controls) with 141 UVCB extracts as described in Section 2, performed in duplicate for each dilution, and compared to ~45 vehicle controls. The TempO-Seq probe sets used herein interrogate the transcription of ~2,900 (~2,500 after collapsing to gene level and low-count removal) expressed transcripts, and the entire experiment provided ~28 million expression data points. The TempO-Seq preprocessing and analysis closely followed the pipeline of House et al. (2017), producing raw sequencing counts that were then subjected to quality control as described in Section 2, followed by normalization by DESeq2 (Love et al., 2014). Differential expression analysis via DESeq2 was performed using both logarithmic fold-change of maximum dose vs. control (“log_2_ FC”), as a simple robust contrast, and a concentration-response trend test using controls and all concentrations, which was expected to be more powerful for monotonic dose-response relationships (Leuraud and Benichou, 2001). Finally, data visualization and various summaries and pathway analyses were used to interpret the biological context.

PCAs of thousands of genes provide a rich empirical visualization environment to examine the overall gene expression pattern relationships in vehicle, media, and DMSO controls (Fig. 1B). In most of the six cell types, the control gene expression patterns are overlapping, suggesting little difference among the control types. This relatively even mixing occurred even when some variational patterns emerged, e.g., for A375s with a small portion of outlying controls, and in the elongated pattern emerging from endothelial controls. For iCell cardiomyocytes, the media controls appear to be somewhat different from vehicle and DMSO. However, our later conclusions are supported across multiple cell types, and we concluded that vehicle controls are appropriate for differential expression analysis. Table S1^1^ shows the average number of reads per expressed transcript for each cell type and treatment, including the three control types. The combinations were roughly comparable in average sequence counts, except that iCell neurons had relatively lower average counts (~300 reads per transcript/probe vs. 500–700 for other cell types).

**Fig. 2:**
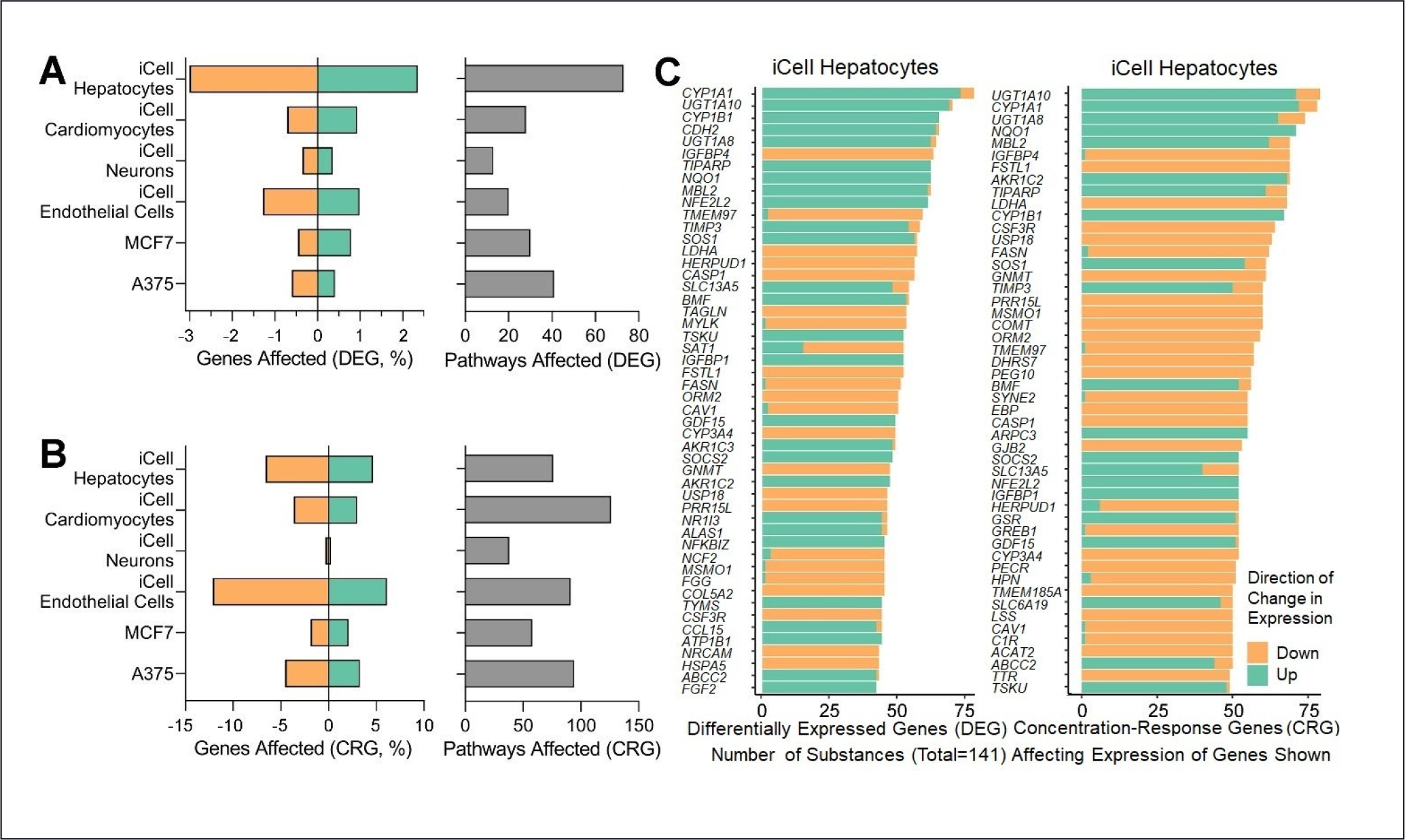
Cell type-specific transcriptional responses to petroleum substances (A) Differentially expressed genes (DEG, DeSeq2 analysis and false discovery *q* ≤ 0.01) were derived by comparing expression between the highest concentration of each substance (*n* = 2) with that of vehicle-treated cells (*n*~45) for each expressed gene. Data are shown as average percent (across the 141 substances) of the total number of genes expressed in each cell type for up- (green) and down- (orange) regulated genes. Pathways that were significantly (false discovery *q* ≤ 0.1) affected among these genes were derived using the Reactome database in the xgr package. (B) Same as (A) but concentration-response genes (*n* = 2 for each concentration and ~45 vehicle-treated) using DeSeq trend analysis, (CRG, false discovery *q* ≤ 10%) are shown. (C) Example of cell-specific (iCell hepatocytes) effects of petroleum substances. Top 50 genes that were affected (up- or downregulated), ranked by the number of substances that had a significant (false discovery *q* ≤ 0.1) effect in either DEG (left) or CRG (right) analysis. The same data as shown in (C) for each cell type are in Figures S1 and S2^1^.

Differential expression analysis was performed on the 141 substances for the six cell types. To judge overall cell type-specific transcriptional responses, we recorded the numbers of DEGs for the fold-change analysis (based on fold change and false discovery cut-offs). iCell hepatocytes showed the most DEGs (~2%–3% in each direction), followed by cardiomyocytes and endothelial cells (Fig. 2A). The number of significant Reactome pathways (across all substances) followed a similar pattern, with ~75 significant pathways for hepatocytes. Overall, as expected, a somewhat larger number of CRGs were significant (*q* < 0.1), and here endothelial cells showed many more CRGs (Fig. 2B), while cardiomyocytes showed more significant Reactome pathways. In aggregate, hepatocytes, cardiomyocytes, and endothelial cells appeared to be the most dose-responsive cell types across the substances. Hepatocytes, in particular, demonstrated consistent effects on gene expression, and we use this cell type as an exemplar for several of the main figures in this manuscript. Figure 2C shows individual genes in hepatocytes that had the largest number of instances of differential and concentration-responsive outcomes (*q* < 0.1) across the 141 substances. The top five DEGs were *Cyp1A1*, *UGT1A10*, *Cyp1B*, *CDH2*, and *UGT1AB* (increasing with dose in most substances). The effects on these genes were highly consistent, appearing as significant in ~50% of the substances. The same data shown in Figure 2C are shown for all cell types in Figures S1 and S2^1^.

**Fig. 3:**
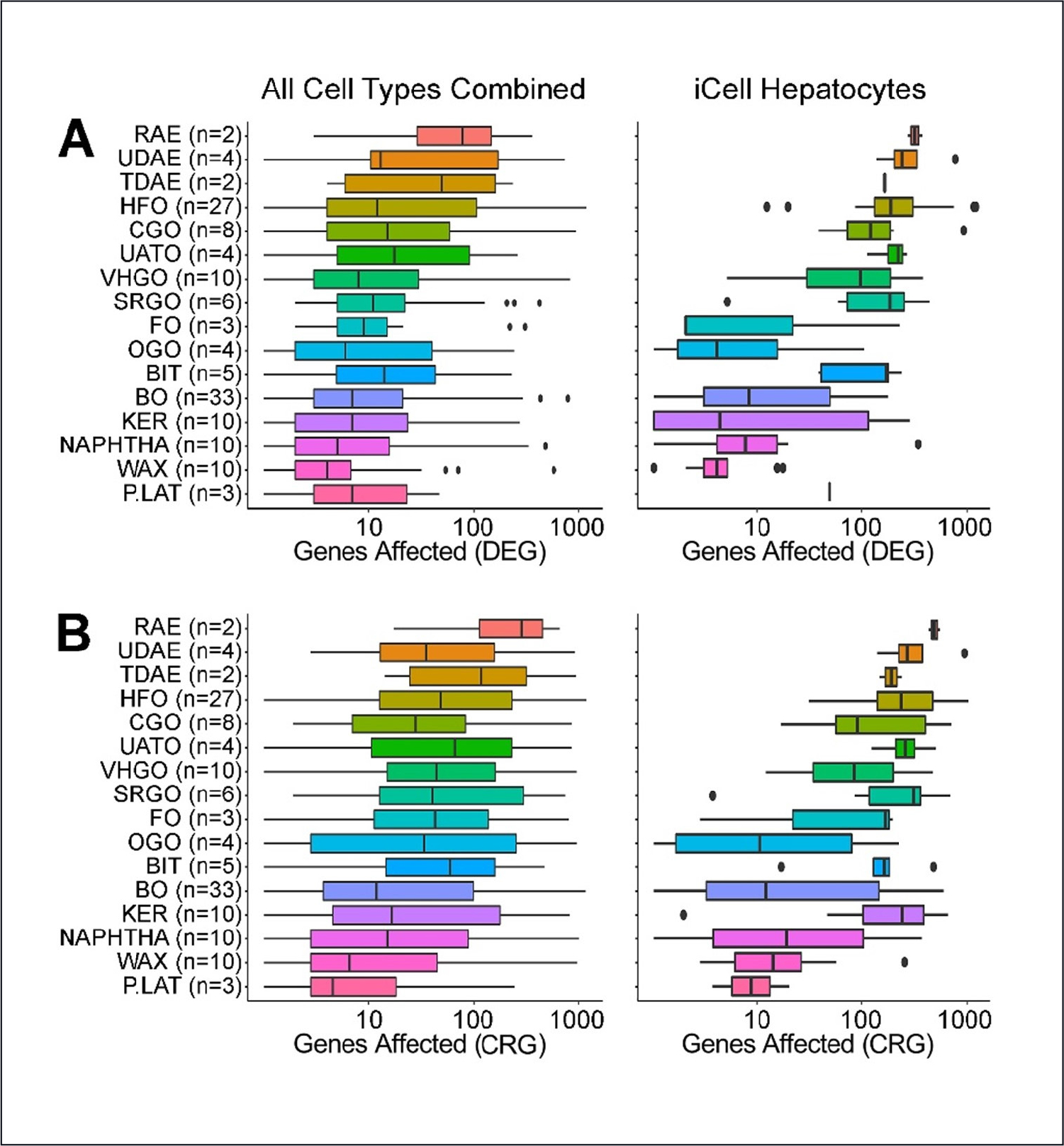
Class-specific effects of petroleum substances on gene expression in the multi-cell *in vitro* transcriptomic analysis Box and whiskers plots show the range in the number of genes significantly (false discovery *q* ≤ 0.1, DeSeq2 analysis) affected by the substances in each class (numbers in each class shown as n). (A) Differentially expressed genes (DEG) were derived by comparing expression between the highest concentration of each substance (*n* = 2) with that in vehicle-treated cells (*n* ~45). (B) Concentration-response genes (CRG) were derived by analyzing the slope in gene expression trend with increasing concentration (*n* = 2 for each concentration and ~45 vehicle-treated). Shown are effects in all cell types (left panels) or in iCell hepatocytes (right panels). Data for each cell type are shown in Figures S3 and S4^1^.

Next, we considered the number of DEGs and CRGs for each substance in each pre-defined manufacturing category. These values are displayed in Figure 3 after aggregating across all cell types (left panels), with a category ordering based on the mean DEG/CRG ranking across all cell types and the categories displaying the most transcriptomic perturbations at the top. Note that a few substances did not show DEG/CRGs after correction for multiple comparisons. The results for hepatocytes are shown in the right panels and are somewhat more variable, both within and across categories, as they are based on a single cell type. However, the correspondence with the bioactivity-based ordering is still apparent, with some exceptions. For example, bitumens, SRGO, and petrolatum appear to have a relatively greater number of DEGs in hepatocytes than might be expected from the all-cell types results. The overall results and ordering show that transcriptomic responsiveness is similar to prior knowledge of manufacturing category, based on analytic properties and previously measured bioactivity (House et al., 2021).

**Fig. 4:**
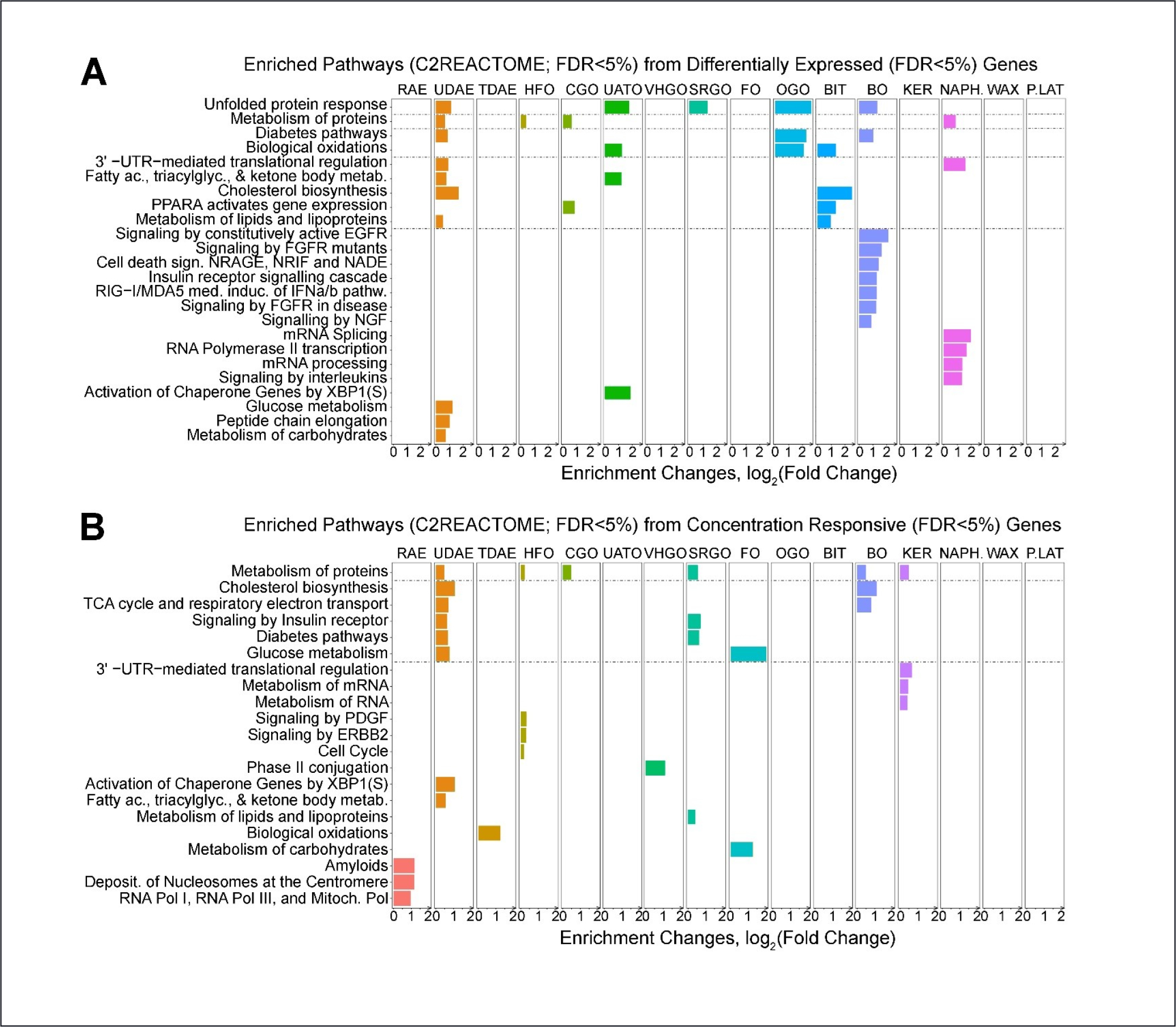
Class-specific effects of petroleum substances on pathway enrichment (xgr package) in gene expression data from iPSC-derived hepatocytes A false discovery *q* threshold of 0.05 was used for the gene set selection. For pathway enrichment, another false discovery *q* threshold of 0.05 on the pathway selection was used. Bar plots show enriched pathways (C2Reactome) at FDR ≤ 5% derived using either differentially expressed genes (A, DEG) or concentration-response genes (B, CRG) affected by the substances in each class. In both cases, the gene-level false discovery of q ≤ 0.05 was used. Shown are all substance classes regardless of whether any pathways were enriched. Pathways are ranked by the degree of overlap among classes. The same data for other cell types are shown in Figures S5 and S6^1^.

Pathway analyses for enrichment of DEGs in Reactome pathways was performed within each cell type and for each manufacturing category. The results for iCell hepatocytes are shown in Figure 4A for DEGs, expressed as the enrichment fold change by the xgr package. The most commonly perturbed pathways, across multiple categories, included “unfolded protein response,” “metabolism of proteins,” “diabetes pathways”, and “biological oxidations.” Another set of related pathways appeared, involving fatty acid metabolism, cholesterol biosynthesis, and PPARα activation. We note that our approach to pathway enrichment requires a contrast between significant genes and the remaining background interrogated set of genes, so that for example the RAE category shows few significant pathways although the number of DEGs is large as previously shown. Similar patterns and results occurred for the CRGs (Fig. 4B).

**Fig. 5:**
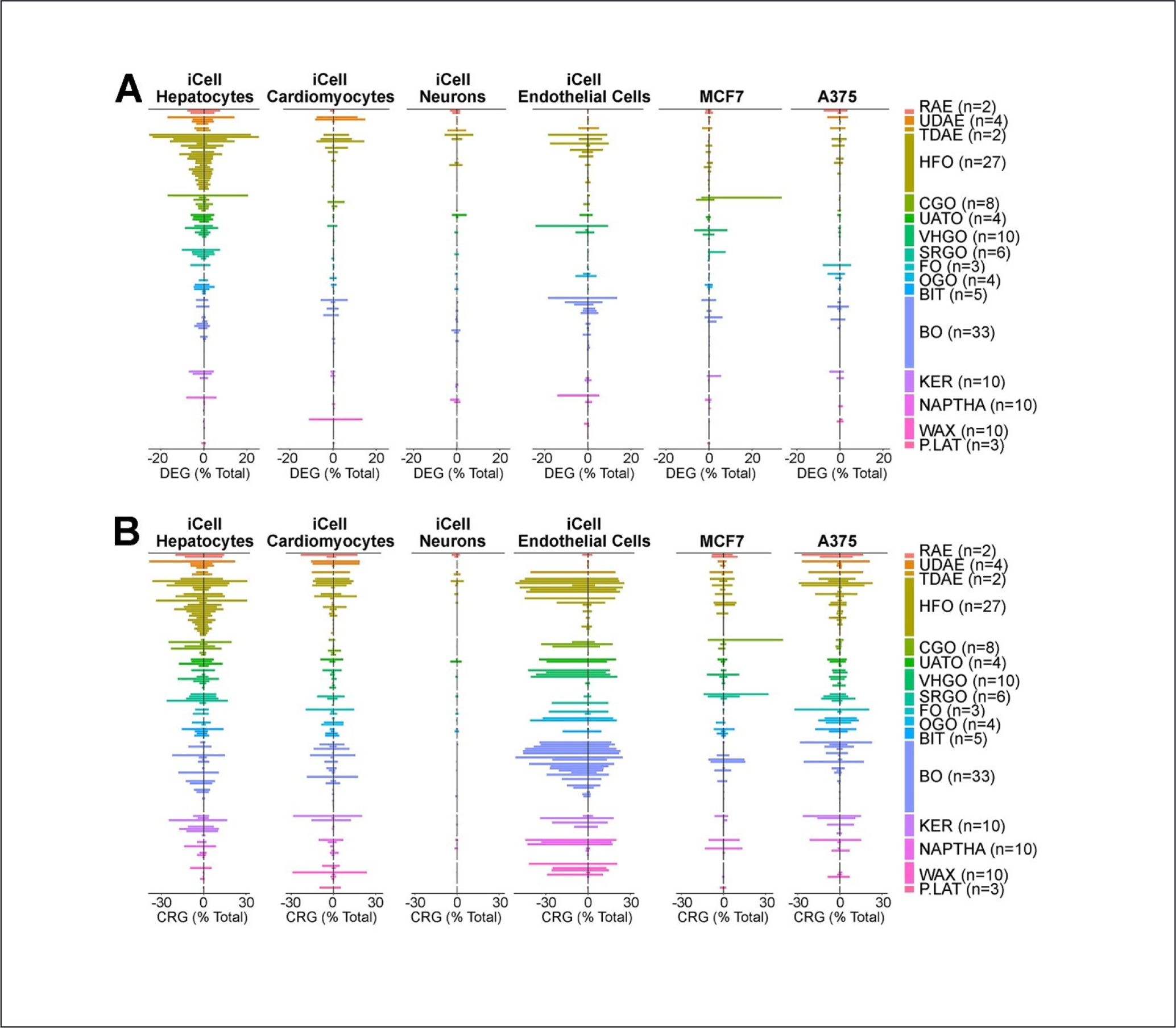
Heterogeneity of the effects of petroleum substances in the multi-cell *in vitro* transcriptomic analysis – cell-specific effects Two-sided bar plots show the percent of genes significantly (false discovery *q* ≤ 0.1) affected (either up- or downregulated) by the substances in each class (numbers in each class shown as *n*). Within class, substances are ranked (top to bottom) based on their cumulative effect across all 6 cell types. (A) Differentially expressed genes (DEG) were derived by comparing expression between the highest concentration of each substance with that in vehicle-treated cells. (B) Concentration-response genes (CRG) were derived by analyzing the slope in gene expression trend with increasing concentration.

The gene expression effects for individual substances, organized by category, are shown in Figure 5A (DEGs) and 5B (GRGs), displaying the number of significant genes per substance (*q* < 0.1). Again, among the cell types, hepatocytes, cardiomyocytes, and endothelial cells showed the greatest effects of UVCBs on gene expression. Direction of expression effects (increase/decrease with increasing concentration) were approximately balanced, except for endothelial cells, which displayed a preponderance of downregulated genes. The number of significant CRGs was generally higher than for DEGs, which we attribute to the greater power of the trend analyses in which all concentrations are used.

**Fig. 6:**
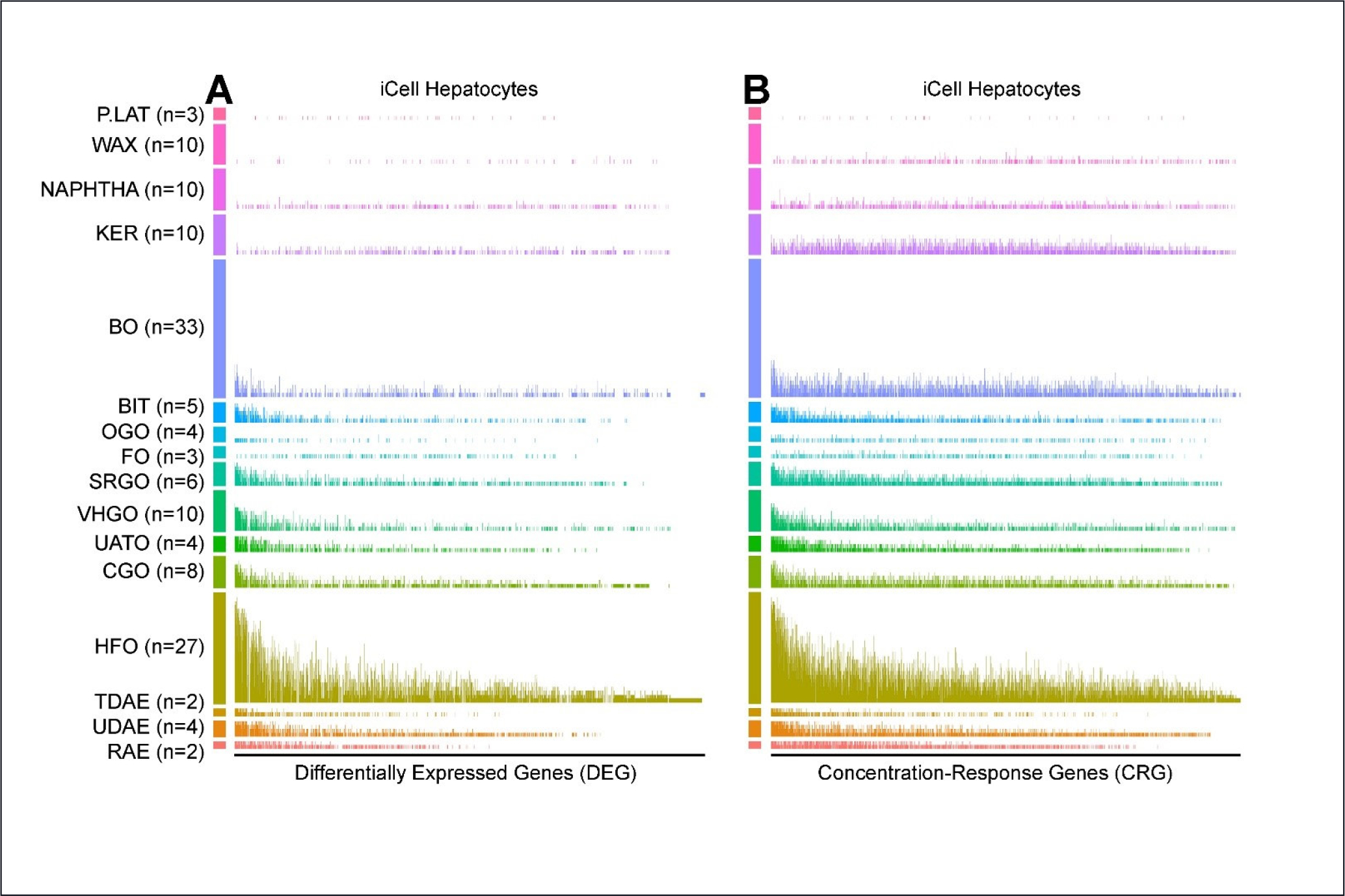
Heterogeneity of the effects of petroleum substances in the multi-cell *in vitro* transcriptomic analysis In the hepatocyte example, 2388 genes were assessed for DEGs and CRGs (false discovery *q* < 0.1) after removal of low-count genes. Each vertical line represents one of these genes, ordered left to right as genes most perturbed across all 141 evaluated substances. The height of each bar represents the proportion of times within the UVCB class (numbers in each class shown as *n*) the gene was either differentially expressed (A) or exhibited a concentration response (B).

Heterogeneity of differential gene expression patterns can also be viewed by considering, for each gene, the number of substances within the manufacturing category in which the gene was significant. Figure 6A shows these results for DEGs (*q* ≤ 0.1) in iCell hepatocytes, with genes ordered according to decreasing number of times perturbed across all substances. The height of each bar represents the proportion of times that gene was perturbed for the given category or class. For manufacturing categories with large numbers of significant genes (such as HFOs), some groups of genes are differentially expressed in most substances within the category. For CRGs (Fig. 6B), the patterns are even clearer, with the plots appearing “denser” due to the larger number of significant genes. Due to the uniform ordering of genes across all test substances, an approximate concordance can be discerned among the categories of high bioactivity (lower on the plots), with the leftmost genes showing the greatest evidence of differential expression as a common feature across multiple categories. For categories of lower bioactivity, the genes showing the most evidence of differential expression are dispersed more evenly throughout, as exemplified by bitumens and the kerosene substances.

**Fig. 7:**
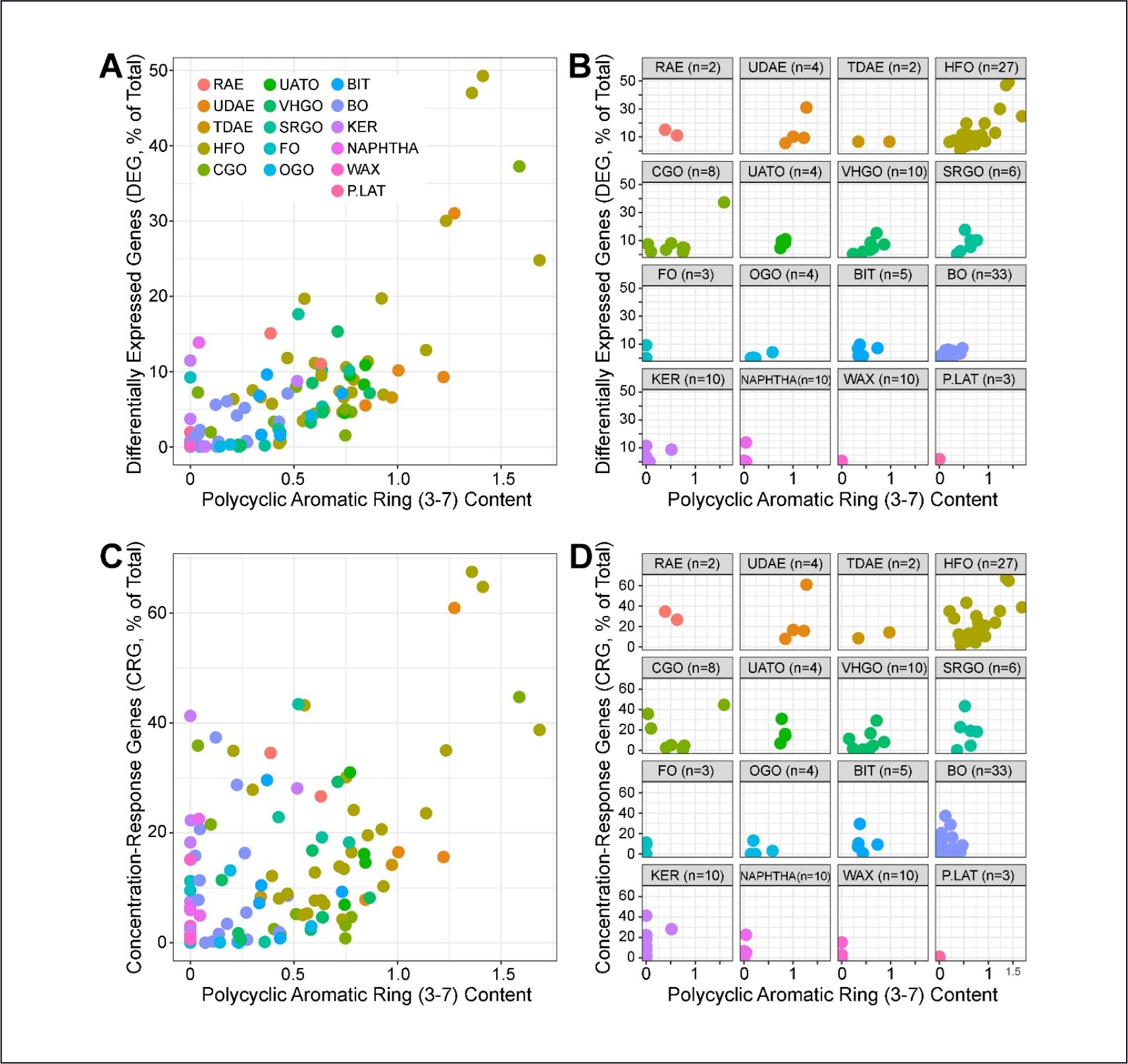
Correlation between the number of DRGs (A,B) or CRGs (C,D) with the extractable 3–7 polycyclic aromatic ring content (PAH) in iCell hepatocytes with FDR ≤ 10% Spearman’s ρ = 0.77 (A) and 0.57 (C).

In a manner similar to results presented in House et al. (2021), we reasoned that substances with higher content of 3–7 ring PAC may elicit more prominent gene expression changes. For each cell type, the number of DEGs and CRGs was compared to the PAC 3–7 content across the 141 UVCBs. Among the cell types, iCell hepatocytes showed the highest correlation (Spearman ρ = 0.77) with DEGs (Fig. 7A) and CRGs (Fig. 7C). Subplots in Figures 7B and 7D show the results within each manufacturing category. It is notable that the positive relationship is discernable even within manufacturing category, provided the category spans a sufficient range of PAC content, as can be observed for HFOs and CGOs. The results of this analysis provide an anchoring to a known aspect of substance hazard for petroleum UVCBs (McKee et al., 2015, 2018). Moreover, the results provide a clear criterion for which cell type, in this case iCell hepatocytes, might be selected for future investigation over other cell types. PAC correlations (Spearman and Pearson) and p-values for each cell type and either DRGs or CRGs are summarized in Table 2.

Finally, we investigated the extent to which machine learning models can be trained to recognize features that are representative of a manufacturing category. Although bioactivity as reported in House et al. (2021) is not the primary subject of this report, the ability of these data to support machine learning analyses can be initially motivated by comparison of expression to bioactivity. A quantitative summary of the 42 bioactivity predictors (House et al., 2021) was used as a response for a cross-validated regression tree model using gene expression within each cell type (see Methods). The resulting correlations for prediction vs. observed bioactivity were: A375 (*r* = 0.81), iCell cardiomyocytes (*rc* = 0.61), iCell endothelial (*r* = 0.76), iCell hepatocytes (*r* = 0.84), MCF7 (*r* = 0.68), and iCell neurons (*r* = 0.35).

**Fig. 8:**
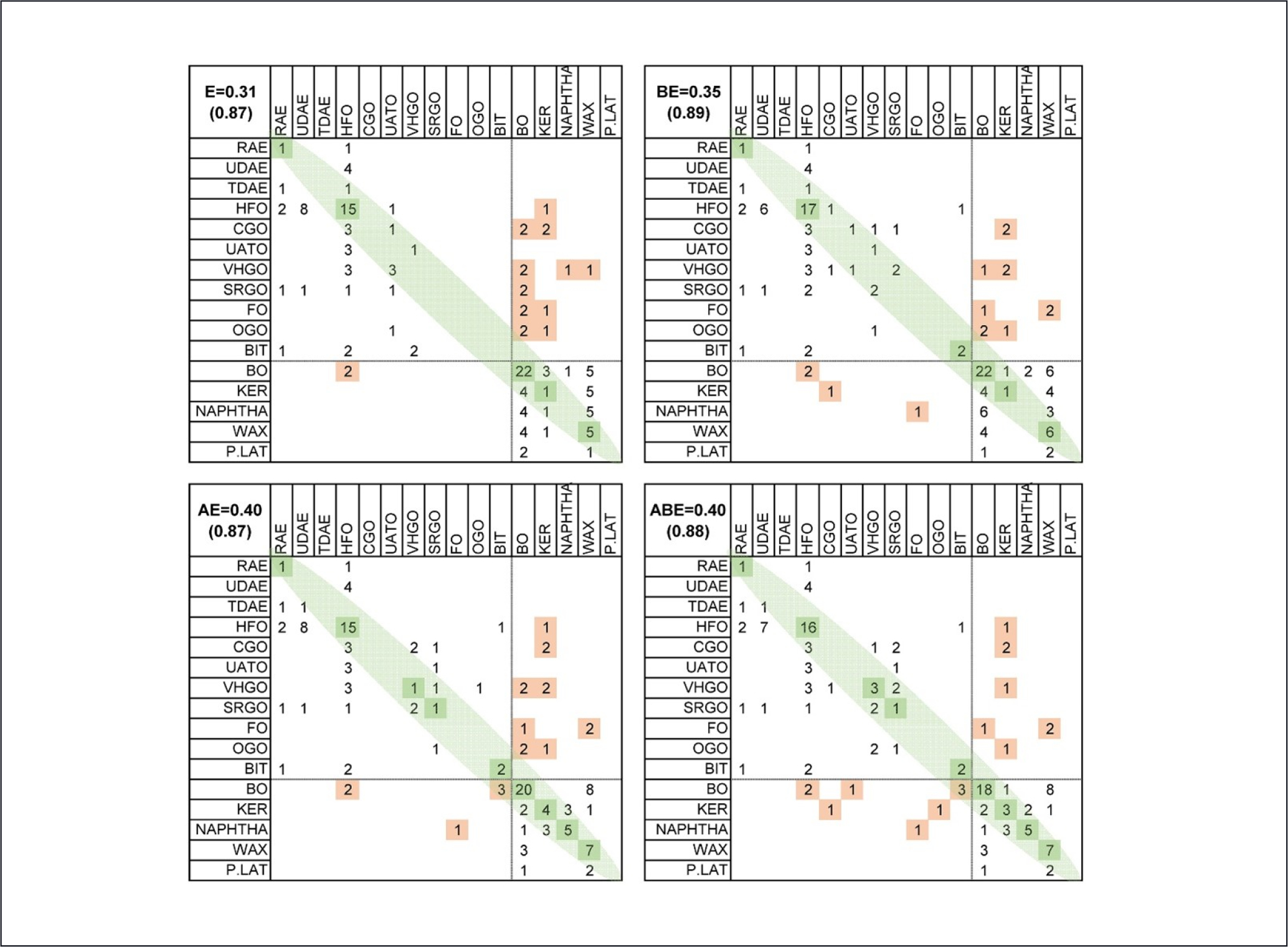
Analysis of the relationship between gene expression data (iCell hepatocytes, “E”), analytical data (PAC, “A”), and summarized bioactivity data (from House et al., 2021, “B”) Top left (E): The results of supervised analysis in which the UVCB category is predicted from the pattern of gene expression data in iCell hepatocytes using the PAM classification procedure as described in Section 2. Rows refer to the true category and columns to predicted category. Correct classification counts are colored in green as values on the diagonal. Categories are ordered according to median bioactivity score, so misclassifications near the diagonal (not colored) are not severe, while misclassifications into categories with substantially different hazard profiles are colored in orange. Top right (BE): correct classifications and misclassifications using both expression and bioactivity patterns. Bottom left (AE): correct classifications and misclassifications using both expression and analytic data. Bottom right (ABE): correct classifications and misclassifications using expression, bioactivity, and analytic data. Numbers in top left corner of each matrix are correct classification rate when only exact matches are considered or (in parenthesis) when misclassifications fall into a hazard category that is not substantially different (P.LAT to BO, or BIT to RAE).

These high correlations in these results support the potential informativeness of these data for categorization and further support iCell hepatocytes as the most informative cell type investigated for expression. Such a “supervised” analysis of categories can potentially provide information about the uniformity of substances in a manufacturing category, as well as highlight substances that are difficult to group within the category and are therefore a priority for future testing or to serve as prioritized group representatives. Such substances may be difficult to identify with unsupervised analyses, which use all the features available (e.g., thousands of genes) and where uninformative feature variation can overwhelm the inference. For this analysis, we used a software originally designed for gene expression class prediction (Tibshirani et al., 2002), but which can use any quantitative predictors. We used the expression of 2,388 expressed genes in various combinations with the 8 analytic (i.e., PAC) and 42 bioactivity predictors previously described in House et al. (2021) for these 141 substances. The results are shown in Figure 8 using hepatocyte expression. In cross-validated analyses, “exact” matches of the model predictions to actual manufacturing category were 31% for expression alone, 35% for bioactivity + expression, 40% for PAC + expression, and 40% for PAC + bioactivity + expression. Although these values are lower than 50%, they are highly significant in comparison to the null 95% permutation threshold of 16%. Closer examination of the results by manufacturing category ordered by bioactivity (Fig. 8) showed much higher classification accuracy (ranging from 87% to 89%) when grouping substances with similar hazard potential. Here, transcriptomic data appears to provide relatively modest improvement compared to analytic or bioactivity analyses (House et al., 2021), which we attribute to the difficulty in gene expression feature selection from among thousands of genes and to the fact that saturated hydrocarbon constituents that were enriched in the extracts used herein may not elicit substance-specific gene expression changes. Using only expression for prediction leads to more “clumping” of prediction into the large categories such as HFOs and BOs, while the combination of predictor types spreads these predictions across other categories, a phenomenon that can be seen when comparing the diagonals of the upper left and lower right panels.

## 4 Discussion

Assessing the potential human health hazard of UVCB substances and, more specifically, defining a targeted testing strategy that will assist in refining and reducing animal testing is a particularly challenging problem in regulatory decision-making. Hazard characterization based on individual components of complex substances is largely intractable, and limited animal testing data are available for risk characterization. However, ethical and economic considerations indicate a critical need to reduce animal testing (Herrmann et al., 2019); therefore, *in vitro* test-based NAMs are under active consideration as the future of risk assessment (Kavlock et al., 2018). Indeed, much work is being performed to collect and catalogue *in vitro* test data on thousands of chemicals (Williams et al., 2017) as well as to demonstrate how they can be used in support of regulatory decision-making (Paul Friedman et al., 2020; Berggren et al., 2015; Chiu et al., 2018; Escher et al., 2019). Far less NAM data is available for complex substances, such as UVCB or environmental mixtures (Drakvik et al., 2020; Bopp et al., 2019).

Previously, we examined the utility of NAMs for grouping of complex UVCBs (House et al., 2021); 141 petroleum-based UVCB substances were grouped based on their biological responses from cell type-specific assays across 15 human cell types. Here, we assessed the informativeness and ability of the transcriptomic data across 6 of these human cell lines to add further mechanistic information to the grouping of these substances. Transcriptomics was among the first omics data types to be used for classification and prediction of hazards and risks of drugs and environmental chemicals (Ganter et al., 2005; Uehara et al., 2010; Waters et al., 2008). While some of the early, over-optimistic forecasts about the value of transcriptomic data for toxicity prediction did not materialize, it has been proposed that these data be routinely collected in toxicology studies and used in risk-based evaluations (Yauk et al., 2020; Liu et al., 2019). Among the most notable developments that support transcriptomics data as a “screening” NAM is the opportunity to conduct high-throughput experiments that interrogate multiple cell-based models and can test for concentration-response in gene expression (House et al., 2017; Phillips et al., 2019). This approach represents a path forward in decision-making, as compared to the traditional use of transcriptomic data to provide mechanistic evidence (Harrill et al., 2019, 2021). Transcriptomic data are high-dimensional and provide a comprehensive set of information on the state of the cells or tissues in both health and disease; this information has been exploited to not only classify individual chemicals with respect to their potential hazard (Ganter et al., 2005; Uehara et al., 2010), but also to group chemicals based on the similarity in their effects (Low et al., 2011; De Abrew et al., 2016, 2019), one of the justifications for grouping and read-across in the regulatory context (Schultz et al., 2015). Finally, we emphasize that transcriptomics provides biological context due to the very nature of genomic annotation that other NAMs (e.g., based on bioactivity as in House et al., 2021) do not so readily provide, and the high adoption rate and standard use of transcriptomics (Yauk et al., 2020; Liu et al., 2019) make it more attractive than the use of NAMs that require highly specialized methods.

In our study, probes from the TempO-seq s1500^+^ gene set (n = 2,982 optimized for human pathway coverage and representative of the human transcriptome) were assessed in replicate across a 4-point concentration dose response. We examined both differential gene expression response at the highest treatment dose compared to controls as well as concentration response effects across all concentrations. The inclusion of gene expression profiling in this study, in addition to the biological response data from the high content *in vitro* screening generated earlier (House et al., 2021), provide (1) biological context to the challenge of grouping of UVCBs with cell-based data, and (2) additional clues as to the suitability of various cell types for screening and prioritization.

For the first challenge, we note that the imaging-based phenotypes on these UVCBs (House et al., 2021) were considered by regulators as insufficient for the purpose of supporting the similarity argument in product registration (ECHA, 2020). It was noted that the relationships between *in vitro* results and *in vivo* effects of these substances needed to be clarified, especially if the registration argues that PAH constituents present the most bioactive, or worst case, fraction of the test substance. Indeed, assessment of DEGs and CRGs across the cell-line/UVCB treatment space revealed additional mechanistic information. For example, in agreement with the hypothesis that PAH are eliciting the majority of transcriptional changes, iPSC-derived hepatocytes were among the most responsive to UVCB-elicited transcriptomic alterations for both DEGs and CRGs across these 141 petroleum substances, with transcriptomic changes observed in active xenobiotic metabolizing genes. Genes most often up-regulated in response to petroleum UVCBs were downstream of nuclear receptor-activated transcription of xenobiotic metabolism genes (e.g., cytochrome P450s and UDP-glucuronosyltransferases) in response to hydrocarbons (Goedtke et al., 2020). Consistent with previously published results (Grimm et al., 2016), the wax and petrolatum categories elicited the fewest transcriptomic changes, while aromatic extracts elicited the strongest response. These results were apparent not only across a summary of all 6 cell types but were also largely replicated in hepatocytes alone. Thus, these data are informative with respect to the second challenge and the design of future *in vitro* experiments for testing petroleum UVCBs, because our results suggest that iPSC-derived hepatocytes may be an especially useful cell type for profiling complex substances that contain PAC 3–7 constituents. This effect was more apparent for DEGs (Spearman’s ρ = 0.77 for the aggregate measure vs. PAH 3–7) than for CRGs (Spearman’s ρ = 0.57). The strength of these relationships, using gene expression and iCell hepatocytes alone, is similar to that observed (ρ = 0.81) for a summary of 4 bioactivity assays in iCell hepatocytes (House et al., 2021).

Various aspects of within- and between-category gene expression changes, as well as variation across cell types, have been explored in this study. Many of these differences are apparent in the “experimental expression fingerprints” provided in Figure 5. More highly significant changes are evident in the concentration response analysis than the differential expression (max. dose vs. vehicle control) analysis, which we attribute to increased power in the use of all the concentration data. The data from iCell hepatocytes exhibited similar proportions and distributions of up- and downregulated DEGs/CRGs, increased expression changes in the fuel oil and aromatic extract categories, within-category response-heterogeneity, and nearly absent gene expression changes within the wax and petrolatum categories. The “gene fingerprints” exhibited in iCell hepatocytes in Figure 6 indicate that for many categories that elicit an overall modest transcriptomic response (e.g., base oils; BO), the genes that are differentially expressed across numerous within-category substances tend to be the same as those identified in categories eliciting a stronger response (e.g., HFOs). Collectively, for petroleum UVCBs, we argue that our data are supportive of hepatocytes as the most suitable cell type for screening if a single cell type is used. For other types of complex substances, we recommend that several cell types still be examined (Chen et al., 2020, 2021; Hsieh et al., 2021), but our general approach may serve as a useful model for such investigations.

Our results on supervised grouping of substances into petroleum substance-specific categories indicates that expression patterns can be used to confirm assignments of individual substances into categories with an accuracy that is much higher than chance (for exact category matches, 38% vs. 16%), but that this classification is more effective if performed using a combination of expression with other bioactivity/analytic data that had been reported previously (House et al., 2021). As noted earlier, one difficulty with expression-based classification is posed by the large number of potential classifiers, which can produce overfitting and reduce cross-validated accuracy. It is also worth noting that existing categorization is based on manufacturing processes that may have an imperfect relationship with biological response. For example, while the correlation between bioactivity and expression vs. PAH 3–7 content is relatively high (House et al., 2021) (Fig. 7), the within-category range for PAC values is large, and categories are not monolithic. Thus, the accuracy of the transcriptomic and *in vitro* assays in general for supervised classification may have upper bounds that do not reflect on the assays themselves.

One additional consideration with respect to the study design and the potential use of these data in support of testing proposals and read-across hypothesis concerns the choice of the DMSO extraction (ASTM International, 2014) to enable *in vitro* testing of petroleum UVCBs. This method preferentially extracts 3–7 ring PAC, but the regulators have noted that “*testing DMSO extracts does not provide a basis for reliably predicting the properties of the [whole] substance*” (ECHA, 2020). Unfortunately, testing the material which is left after DMSO extraction presents numerous challenges because the solvents that would need to be used are incompatible with *in vitro* testing. Alternative delivery of the “whole substance” can be achieved through passive dosing (Hammershoj et al., 2020; Trac et al., 2021); however, the methods to deliver complex UVCBs in small volume *in vitro* testing conditions have not been established yet. Thus, additional work is needed to improve the relevance of *in vitro* test methods for use in regulatory decisions on UVCBs.

## Supplementary Material

Supplemental materials

## Figures and Tables

**Tab. 1:** Petroleum substance categories and substances used in this study See Table S1^1^ of House et al. (2021) for a complete listing of substance names, CAS and EC numbers, and other information.

Petroleum substance category	Category abbreviation	N of samples in category
Residual aromatic extracts	RAE	2
Untreated distillate aromatic extracts	UDAE	4
Treated distillate aromatic extracts	TDAE	2
Heavy fuel oil components	HFO	27
Cracked gas oils	CGO	8
Unrefined/acid treated oils	UATO	4
Vacuum gas oils, hydrocracked gas oils & distillate fuels	VHGO	10
Straight-run gas oils	SRGO	6
Foots oils	FO	3
Other gas oils	OGO	4
Bitumens/oxidized asphalt	BIT	5
Other lubricant base oils/highly refined base oils	BO	33
Kerosines/MK1 diesel fuel	KER	10
Low boiling point naphthas (gasolines)	NAPHTHA	10
Paraffin and hydrocarbon waxes/slack waxes	WAX	10
Petrolatums	P.LAT	3

**Tab. 2:** Cell-specific relationships between gene expression effects (either as differentially expressed genes, DEG; or concentration-response genes, CRG) and polycyclic aromatic compound (PAC, 3–7 ring) content of petroleum UVCBs tested in this study Both Pearson *r* and Spearman *ϸ* are shown with corresponding *p*-values for each correlation. See Figure 7 for cell type-specific correlation plots.

Cell type	Type	*r*	*p*-value	*ρ*	*p*-value
iCell hepatocytes	DEG	0.77	1.8E-26	0.76	5.9E-25
	CRG	0.57	2.6E-12	0.55	1.9E-11
iCell cardiomyocytes	DEG	0.57	1.0E-12	0.16	0.071
	CRG	0.21	0.014	0.14	n.s.
iCell neurons	DEG	0.39	1.4E-05	0.07	n.s.
	CRG	0.20	0.047	0.15	n.s.
iCell endothelial	DEG	0.13	n.s.	0.06	n.s.
	CRG	0.09	n.s.	0.08	n.s.
MCF7	DEG	−0.04	n.s.	0.26	0.005
	CRG	−0.04	n.s.	0.25	0.006
A375	DEG	0.11	n.s.	0.08	n.s.
	CRG	0.19	0.026	0.32	2.1E-04
All cells combined	DEG	0.34	5.9E-21	0.24	5.2E-11
	CRG	0.16	8.9E-06	0.23	1.4E-10

## Data Availability

MIAME-compliant expression data and experimental metadata have been uploaded to the public repository Gene Expression Omnibus (GSE186121).

## Footnotes

1 doi:10.14573/altex.2107051s
