# Supplementary material for: Grouping of UVCB Substances with Dose-Response Transcriptomics Data from Human Cell-Based Assays
Source: ALTEX. Author manuscript; Available in PMC 2022 Aug 2. (PMC9344966; doi:10.14573/altex.2107051)
Supplement: Supplemental materials [file NIHMS1827292-supplement-Supplemental_materials.pdf]

House et al.:

Grouping of UVCB Substances with Dose-Response Transcriptomics Data from Human Cell-Based Assays

Supplementary Data

Tab. S1: Average number of reads per expressed transcript for each cell type and treatment combination
Please see Section 2 for explanation of the vehicle, media, and DMSO concentrations for each cell type. Each test substance was examined in 3 concentrations ("Conc.") with "100" being the most concentrated and "10" and "1" being 10-fold subsequent dilutions.

Cell type	Organ	Origin	Vehicle	Media	DMSO	Conc. 1	Conc. 10	Conc. 100
iCell Hepatocytes	Liver	iPSC-derived	639	589	612	557	531	546
iCell Cardiomyocytes	Heart	iPSC-derived	721	729	660	641	484	442
iCell Neurons	Brain	iPSC-derived	381	409	381	248	453	337
iCell Endothelial	Blood vessel	iPSC-derived	521	434	393	355	424	321
MCF7	Breast	Epithelial adenocarcinoma	562	599	550	586	423	485
A375	Skin	Malignant melanoma	733	760	689	704	569	685

Supplementary description of cell culture conditions

iCell Hepatocytes 2.0
Vials of hepatocytes were thawed for 3 min at 37°C in a water bath and subsequently resuspended in RPMI medium containing 2% (v/v) iCell hepatocyte medium supplement, 0.1 µM dexamethasone, 2% (v/v) B27 supplement, 25 µg/mL gentamicin, and 20 ng/mL oncostatin-M. Following microscopic evaluation of the cell density, the suspension was further diluted to a final concentration of 6.72 × 10^5 cells/mL. 25 µL of this suspension was then added to each well on collagen I coated 384-well plates (Corning, Product# 354664), yielding a final cell density of 16,800 cells per well. Plates were initially kept at room temperature (RT) for 30 min and then transferred to an incubator set at 37°C and 5% CO_2. After 4 h of incubation, the plating medium was replaced with 25 µL fresh medium, a step that was repeated daily for 4 days. On day five, the plating medium was exchanged with 25 µL per well maintenance medium, consisting of RPMI containing 2% (v/v) iCell hepatocyte medium supplement, 0.1 µM dexamethasone, 2% (v/v) B27 supplement, and 25 µg/mL gentamicin. Maintenance medium was exchanged daily for the duration of the experiment. See additional details in Grimm et al. (2015).

iCell Neurons
Cryopreserved cells were thawed and plated according to the protocol provided by Cellular Dynamics International. Briefly, cells were plated on poly-D-lysine precoated 384-well plates (Greiner-Bio, Ref#: 781946) with iCell Neural Base Medium (Catalog#: M1010) added with iCell Neural Supplement A (Catalog#: M1032) and 3.3 mg/mL of laminin. Cells were plated at densities of 7,500 cells/well. Plates were initially kept at RT for 30 min before transferring to an incubator set at 37°C and 5% CO_2 for 48 h until assay day. See additional details in Grimm et al. (2015).

iCell Cardiomyocytes
384-well microplates were precoated with 25 µL 0.1% (w/v) gelatin solution per well for 2 h at 37°C and 5% CO_2. Cryopreserved cells were thawed according to the manufacturer's instruction using iCell cardiomyocyte plating medium with 1:500 (v/v) penicillin/streptomycin. Cell suspension was diluted in plate medium to provide a final cell concentration of 2 × 10^5 cells/mL. Subsequently, the gelatin solution was aspirated from the plates and 25 µL cell suspension was added to each well, making the final cell plating density 5,000 viable cells/well. Plates were kept at room temperature for 30 min before they were incubated at 37°C and 5% CO_2. 48 h following cell seeding, the plating medium was exchanged with 40 µL of maintenance medium containing 1:500 penicillin/streptomycin. Maintenance medium was subsequently changed every other day for another 12 days until assay day. See additional details in Grimm et al. (2016).

doi:10.14573/altex.2107051s

This is an Open Access article distributed under the terms of the Creative Commons Attribution 4.0 International license (http://creativecommons.org/licenses/by/4.0/), which permits unrestricted use, distribution and reproduction in any medium, provided the original work is appropriately cited.

iCell Endothelial cells

Endothelial cells were plated and expanded on T-75 tissue culture flasks coated with human fibronectin solution at 3 µg/cm^2. Cells were cultured with maintenance medium containing the VascuLife VEGF Medium Complete Kit (SKU: LL-0003), with FBS, and iCell Endothelial cells medium supplement. Cell density was determined using Trypan Blue exclusion test, and a cell suspension was prepared that resulted in 1.0 × 10^4 cells/cm^2. The fibronectin solution was aspirated and cells were seeded in a T-75 flask. Cells were incubated at 37°C and 5% CO_2 with media changes every 2 days and passaged every 3-4 days by TrypLE Express.

Experiments were conducted with cells between passages 1 and 5. Cells were transferred into 384-well plates with 50 µL maintenance medium at a density of 750 cells/well for cytotoxicity assay and 7,500 cells for angiogenesis assay. Cells were kept in microplates for 2-3 days until a monolayer formed before adding chemicals for cytotoxicity assays. See additional details in Iwata et al. (2017)

A375 and MCF7

Cell lines were obtained from ECACC. A375 were maintained in DMEM HG (Gibco) without phenol red, 10% HI FBS (1050064 South America Origin), 2 mM L-glutamine, pen/strep 100 µg/mL / 100 U/mL, and split every 5-7 days 1:6. MCF7 were maintained in EMEM (Gibco), 10% HI FBS (1050064 South America Origin), 2 mM NEAA (5 mL), 2 mM L-glutamine, pen/strep 100 µg/mL / 100 U/mL, and split every 7-10 days 1:3. All cell lines were used through maximum 20 passages and then replaced from frozen stock.

For use, the harvest cell suspension was counted using a haemocytometer and diluted for seeding in 384-well plates for treatment. 12,000-14,000 cells/well gave 90% confluence within 2-3 days of seeding in 50µl of the 10% FBS growth medium.

For assay, the medium for growth and maintenance, 50 µL/well, was removed on the morning of the day of treatment and replaced with to 20 µL/well fresh medium without FBS. 200X stock plates for $\log_{10}$ dilutions of the UVCB substances where pure extracted substance is 1X and then dilutions from there of 10X, 100X and 1000X were set up. Positive controls at 200X final concentration were also included in this plate. The UVCB substance 200X with positive control plate was first diluted 40-fold by diluting 4 µL stock chemical with 156 µL fresh medium without FBS. After mixing by trituration and microplate spinning, 5 µL of treatment solution from these diluted plates was added to the 20 µL medial on the cells to give a final 200-fold dilution from the 200X stock. Thus, final concentrations of the UVCB substance extract were 200X, 2000X, 20,000X and 200,000X. Treated plates were incubated for total 24 h and processed according to the individual assay requirements according to the manufacturer's protocols.

References

Grimm, F. A., Iwata, Y., Sirenko, O. et al. (2015). High-content assay multiplexing for toxicity screening in induced pluripotent stem cell-derived cardiomyocytes and hepatocytes. *Assay Drug Dev Technol 13*, 529-546. doi:10.1089/adt.2015.659

Grimm, F. A., Iwata, Y., Sirenko, O. et al. (2016). A chemical-biological similarity-based grouping of complex substances as a prototype approach for evaluating chemical alternatives. *Green Chem 18*, 4407-4419. doi:10.1039/c6gc01147k

Iwata, Y., Klaren, W. D., Lebakken, C. S. et al. (2017). High-content assay multiplexing for vascular toxicity screening in induced pluripotent stem cell-derived endothelial cells and human umbilical vein endothelial cells. *Assay Drug Dev Technol 15*, 267-279. doi:10.1089/adt.2017.786

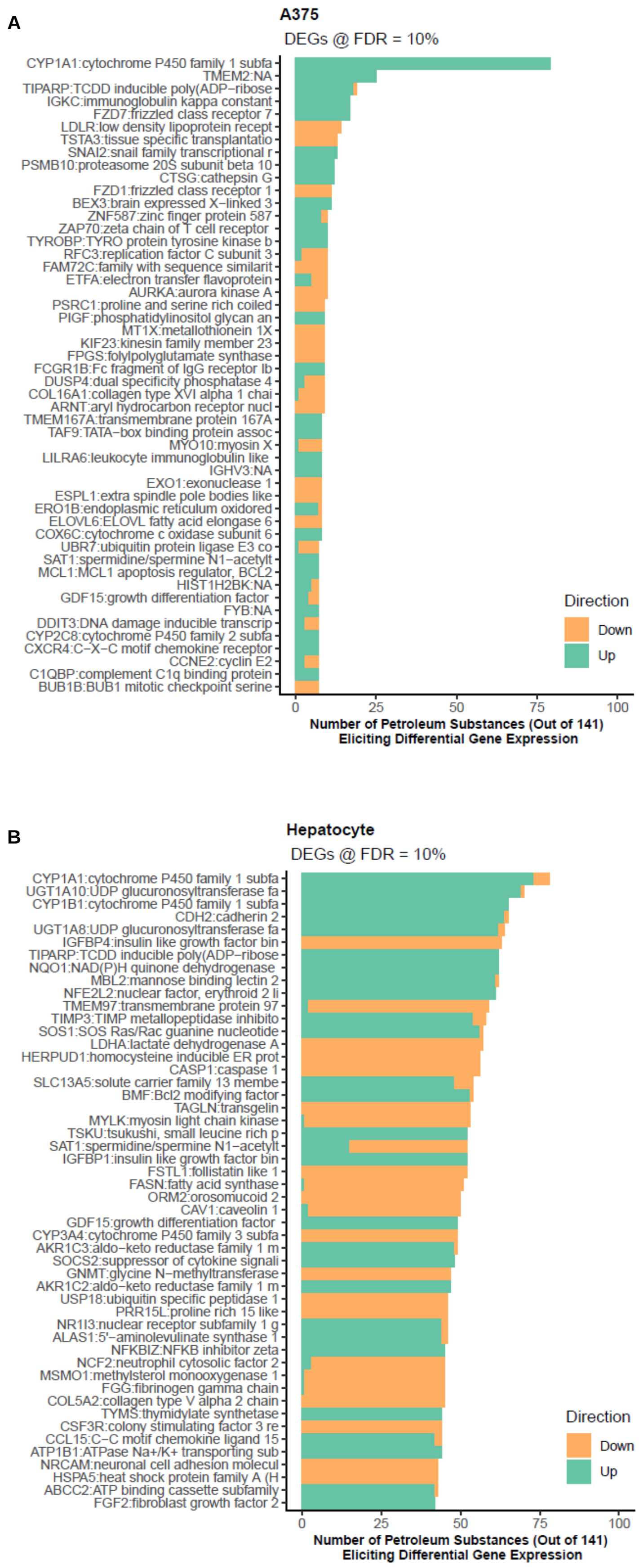

Fig. S1: Top 50 differentially expressed genes by cell type across 141 petroleum substances when comparing the maximum dose to method blank controls

The bar width represents the number of times a given gene was differentially expressed (FDR = 10%) when assessed across each of 141 substances. Orange represents down-regulated gene expression while green represents up-regulation.

C

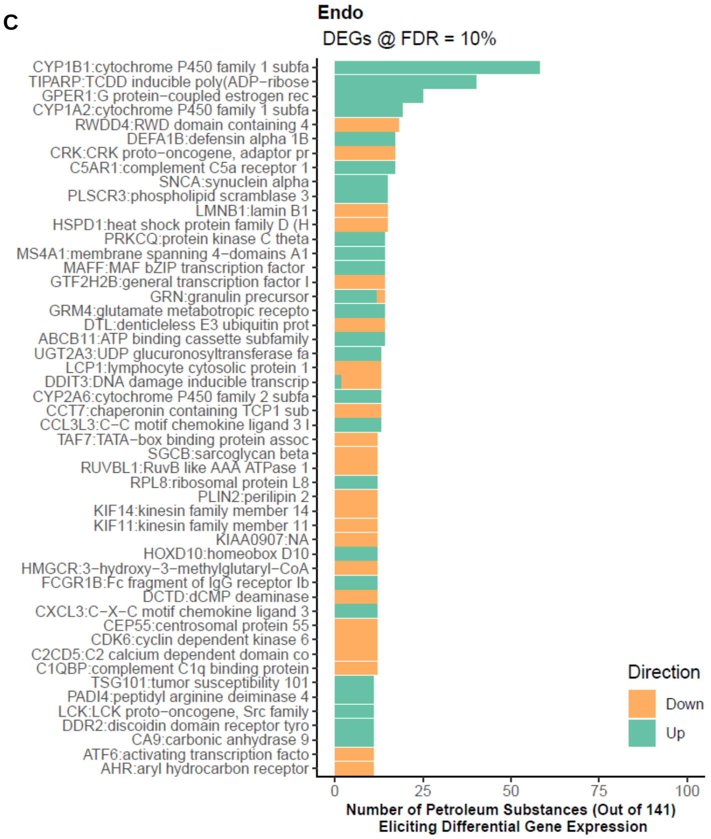
Endo
DEGs @ FDR = 10%
CYP1B1:cytochrome P450 family 1 subfa
TIPARP:TCDD inducible poly(ADP−ribose
GPER1:G protein−coupled estrogen rec
CYP1A2:cytochrome P450 family 1 subfa
RWDD4:RWD domain containing 4
DEFA1B:defensin alpha 1B
CRK:CRK proto−oncogene, adaptor pr
C5AR1:complement C5a receptor 1
SNCA:synuclein alpha
PLSCR3:phospholipid scramblase 3
LMNB1:lamin B1
HSPD1:heat shock protein family D (H
PRKCQ:protein kinase C theta
MS4A1:membrane spanning 4−domains A1
MAFF:MAF bZIP transcription factor
GTF2H2B:general transcription factor I
GRN:granulin precursor
GRM4:glutamate metabotropic recepto
DTL:denticleless E3 ubiquitin prot
ABCB11:ATP binding cassette subfamily
UGT2A3:UDP glucuronosyltransferase fa
LCP1:lymphocyte cytosolic protein 1
DDIT3:DNA damage inducible transcrip
CYP2A6:cytochrome P450 family 2 subfa
CCT7:chaperonin containing TCP1 sub
CCL3L3:C−C motif chemokine ligand 3 l
TAF7:TATA−box binding protein assoc
SGCB:sarcoglycan beta
RUVBL1:RuvB like AAA ATPase 1
RPL8:ribosomal protein L8
PLIN2:perilipin 2
KIF14:kinesin family member 14
KIF11:kinesin family member 11
KIAA0907:NA
HOXD10:homeobox D10
HMGCR:3−hydroxy−3−methylglutaryl−CoA
FCGR1B:Fc fragment of IgG receptor Ib
DCTD:dCMP deaminase
CXCL3:C−X−C motif chemokine ligand 3
CEP55:centrosomal protein 55
CDK6:cyclin dependent kinase 6
C2CD5:C2 calcium dependent domain co
C1QBP:complement C1q binding protein
TSG101:tumor susceptibility 101
PADI4:peptidyl arginine deiminase 4
LCK:LCK proto−oncogene, Src family
DDR2:discoidin domain receptor tyro
CA9:carbonic anhydrase 9
ATF6:activating transcription facto
AHR:aryl hydrocarbon receptor
0
25
50
75
100
Number of Petroleum Substances (Out of 141)
Eliciting Differential Gene Expression
Direction
Down
Up

D

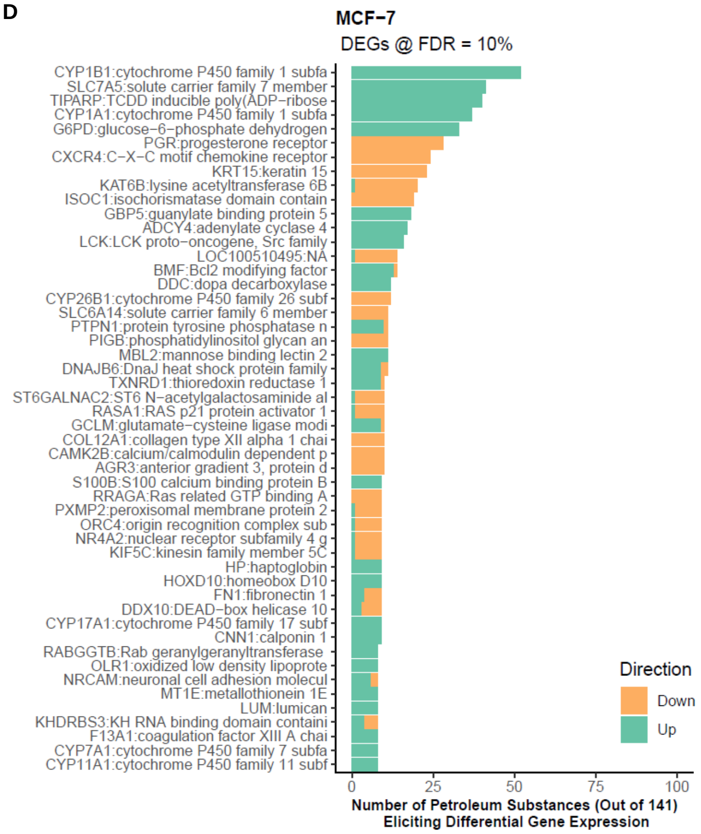
MCF−7
DEGs @ FDR = 10%
CYP1B1:cytochrome P450 family 1 subfa
SLC7A5:solute carrier family 7 member
TIPARP:TCDD inducible poly(ADP−ribose
CYP1A1:cytochrome P450 family 1 subfa
G6PD:glucose−6−phosphate dehydrogen
PGR:progesterone receptor
CXCR4:C−X−C motif chemokine receptor
KRT15:keratin 15
KAT6B:lysine acetyltransferase 6B
ISOC1:isochorismatase domain contain
GBP5:guanylate binding protein 5
ADCY4:adenylate cyclase 4
LCK:LCK proto−oncogene, Src family
LOC100510495:NA
BMF:Bcl2 modifying factor
DDC:dopa decarboxylase
CYP26B1:cytochrome P450 family 26 subf
SLC6A14:solute carrier family 6 member
PTPN1:protein tyrosine phosphatase n
PIGB:phosphatidylinositol glycan an
MBL2:mannose binding lectin 2
DNAJB6:DnaJ heat shock protein family
TXNRD1:thioredoxin reductase 1
ST6GALNAC2:ST6 N−acetylgalactosaminide al
RASA1:RAS p21 protein activator 1
GCLM:glutamate−cysteine ligase modi
COL12A1:collagen type XII alpha 1 chai
CAMK2B:calcium/calmodulin dependent p
AGR3:anterior gradient 3, protein d
S100B:S100 calcium binding protein B
RRAGA:Ras related GTP binding A
PXMP2:peroxisomal membrane protein 2
ORC4:origin recognition complex sub
NR4A2:nuclear receptor subfamily 4 g
KIF5C:kinesin family member 5C
HP:haptoglobin
HOXD10:homeobox D10
FN1:fibronectin 1
DDX10:DEAD−box helicase 10
CYP17A1:cytochrome P450 family 17 subf
CNN1:calponin 1
RABGGTB:Rab geranylgeranyltransferase
OLR1:oxidized low density lipoprote
NRCAM:neuronal cell adhesion molecul
MT1E:metallothionein 1E
LUM:lumican
KHDRBS3:KH RNA binding domain containi
F13A1:coagulation factor XIII A chai
CYP7A1:cytochrome P450 family 7 subfa
CYP11A1:cytochrome P450 family 11 subf
0
25
50
75
100
Number of Petroleum Substances (Out of 141)
Eliciting Differential Gene Expression
Direction
Down
Up

E

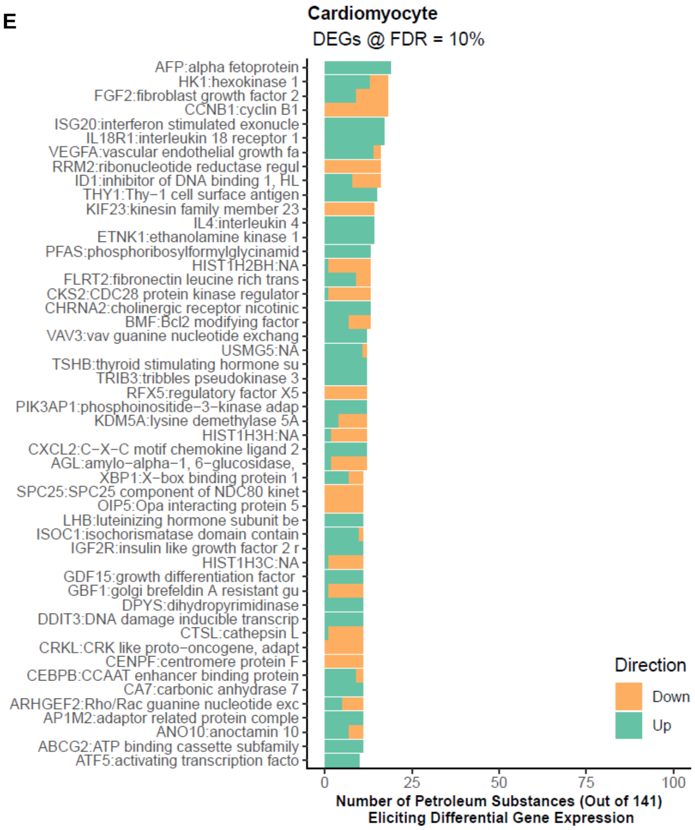
Cardiomyocyte
DEGs @ FDR = 10%
AFP:alpha fetoprotein
HK1:hexokinase 1
FGF2:fibroblast growth factor 2
CCNB1:cyclin B1
ISG20:interferon stimulated exonucle
IL18R1:interleukin 18 receptor 1
VEGFA:vascular endothelial growth fa
RRM2:ribonucleotide reductase regul
ID1:inhibitor of DNA binding 1, HL
THY1:Thy−1 cell surface antigen
KIF23:kinesin family member 23
IL4:interleukin 4
ETNK1:ethanolamine kinase 1
PFAS:phosphoribosylformylglycinamid
HIST1H2BH:NA
FLRT2:fibronectin leucine rich trans
CKS2:CDC28 protein kinase regulator
CHRNA2:cholinergic receptor nicotinic
BMF:Bcl2 modifying factor
VAV3:vav guanine nucleotide exchang
USMG5:NA
TSHB:thyroid stimulating hormone su
TRIB3:tribbles pseudokinase 3
RFX5:regulatory factor X5
PIK3AP1:phosphoinositide−3−kinase adap
KDM5A:lysine demethylase 5A
HIST1H3H:NA
CXCL2:C−X−C motif chemokine ligand 2
AGL:amylo−alpha−1, 6−glucosidase,
XBP1:X−box binding protein 1
SPC25:SPC25 component of NDC80 kinet
OIP5:Opa interacting protein 5
LHB:luteinizing hormone subunit be
ISOC1:isochorismatase domain contain
IGF2R:insulin like growth factor 2 r
HIST1H3C:NA
GDF15:growth differentiation factor
GBF1:golgi brefeldin A resistant gu
DPYS:dihydropyrimidinase
DDIT3:DNA damage inducible transcrip
CTSL:cathepsin L
CRKL:CRK like proto−oncogene, adapt
CENPF:centromere protein F
CEBPB:CCAAT enhancer binding protein
CA7:carbonic anhydrase 7
ARHGEF2:Rho/Rac guanine nucleotide exc
AP1M2:adaptor related protein comple
ANO10:anoctamin 10
ABCG2:ATP binding cassette subfamily
ATF5:activating transcription facto
0
25
50
75
100
Number of Petroleum Substances (Out of 141)
Eliciting Differential Gene Expression
Direction
Down
Up

F

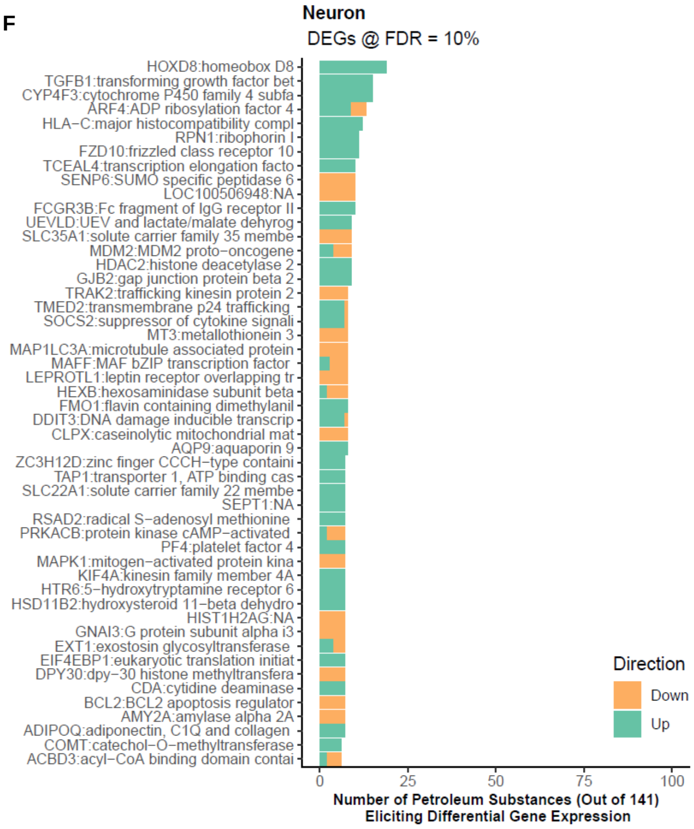
Neuron
DEGs @ FDR = 10%
HOXD8:homeobox D8
TGFB1:transforming growth factor bet
CYP4F3:cytochrome P450 family 4 subfa
ARF4:ADP ribosylation factor 4
HLA−C:major histocompatibility compl
RPN1:ribophorin I
FZD10:frizzled class receptor 10
TCEAL4:transcription elongation facto
SENP6:SUMO specific peptidase 6
LOC100506948:NA
FCGR3B:Fc fragment of IgG receptor II
UEVLD:UEV and lactate/malate dehyrog
SLC35A1:solute carrier family 35 membe
MDM2:MDM2 proto−oncogene
HDAC2:histone deacetylase 2
GJB2:gap junction protein beta 2
TRAK2:trafficking kinesin protein 2
TMED2:transmembrane p24 trafficking
SOCS2:suppressor of cytokine signali
MT3:metallothionein 3
MAP1LC3A:microtubule associated protein
MAFF:MAF bZIP transcription factor
LEPROTL1:leptin receptor overlapping tr
HEXB:hexosaminidase subunit beta
FMO1:flavin containing dimethylanil
DDIT3:DNA damage inducible transcrip
CLPX:caseinolytic mitochondrial mat
AQP9:aquaporin 9
ZC3H12D:zinc finger CCCH−type containi
TAP1:transporter 1, ATP binding cas
SLC22A1:solute carrier family 22 membe
SEPT1:NA
RSAD2:radical S−adenosyl methionine
PRKACB:protein kinase cAMP−activated
PF4:platelet factor 4
MAPK1:mitogen−activated protein kina
KIF4A:kinesin family member 4A
HTR6:5−hydroxytryptamine receptor 6
HSD11B2:hydroxysteroid 11−beta dehydro
HIST1H2AG:NA
GNAI3:G protein subunit alpha i3
EXT1:exostosin glycosyltransferase
EIF4EBP1:eukaryotic translation initiat
DPY30:dpy−30 histone methyltransfera
CDA:cytidine deaminase
BCL2:BCL2 apoptosis regulator
AMY2A:amylase alpha 2A
ADIPOQ:adiponectin, C1Q and collagen
COMT:catechol−O−methyltransferase
ACBD3:acyl−CoA binding domain contai
0
25
50
75
100
Number of Petroleum Substances (Out of 141)
Eliciting Differential Gene Expression
Direction
Down
Up

A

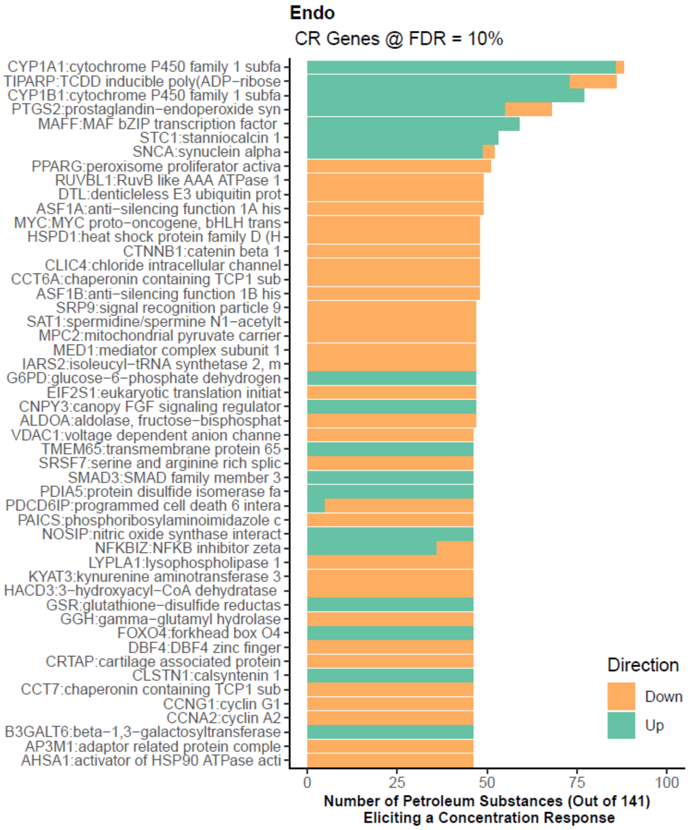

S.Fig. 2B

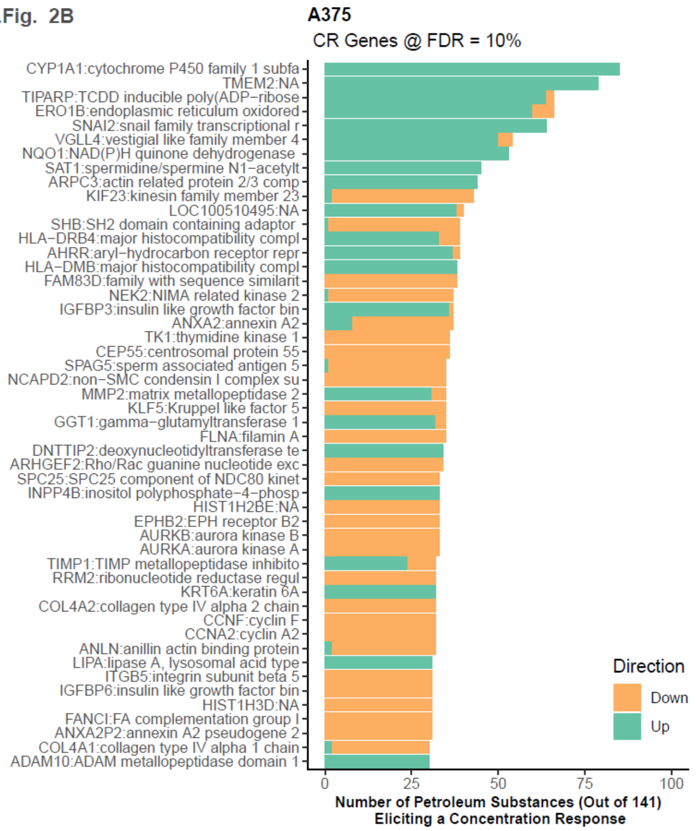

Fig. S2: Top 50 concentration responsive genes by cell type across 141 petroleum substances
The bar width represents the number of times a given gene elicited a concentration response (FDR = 10%) when assessed across each of 141 substances. Orange represents decreasing expression with concentration, while green represents increasing gene expression with concentration.

C

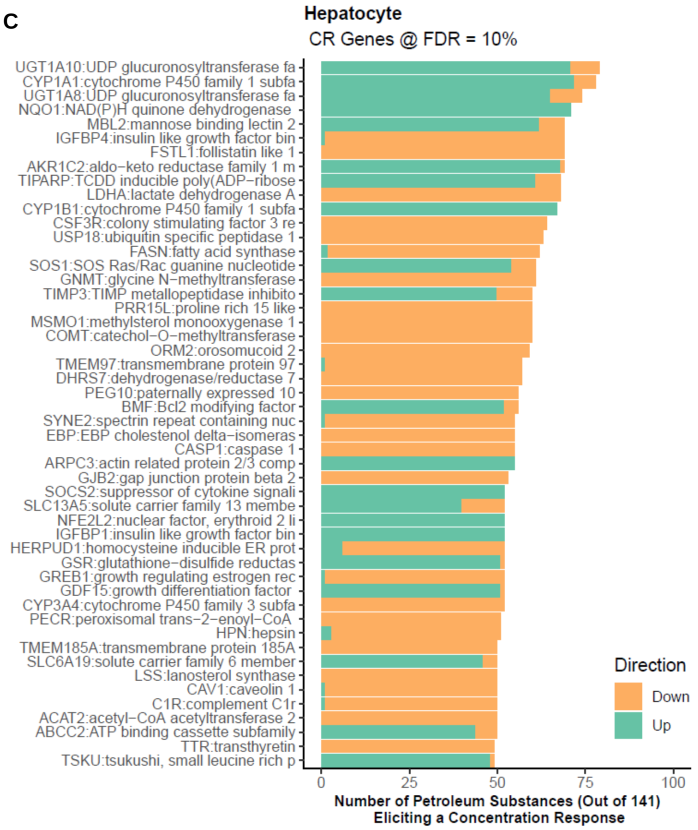
Hepatocyte
CR Genes @ FDR = 10%
UGT1A10:UDP glucuronosyltransferase fa
CYP1A1:cytochrome P450 family 1 subfa
UGT1A8:UDP glucuronosyltransferase fa
NQO1:NAD(P)H quinone dehydrogenase
MBL2:mannose binding lectin 2
IGFBP4:insulin like growth factor bin
FSTL1:follistatin like 1
AKR1C2:aldo−keto reductase family 1 m
TIPARP:TCDD inducible poly(ADP−ribose
LDHA:lactate dehydrogenase A
CYP1B1:cytochrome P450 family 1 subfa
CSF3R:colony stimulating factor 3 re
USP18:ubiquitin specific peptidase 1
FASN:fatty acid synthase
SOS1:SOS Ras/Rac guanine nucleotide
GNMT:glycine N−methyltransferase
TIMP3:TIMP metallopeptidase inhibito
PRR15L:proline rich 15 like
MSMO1:methylsterol monooxygenase 1
COMT:catechol−O−methyltransferase
ORM2:orosomucoid 2
TMEM97:transmembrane protein 97
DHRS7:dehydrogenase/reductase 7
PEG10:paternally expressed 10
BMF:Bcl2 modifying factor
SYNE2:spectrin repeat containing nuc
EBP:EBP cholesterol delta−isomeras
CASP1:caspase 1
ARPC3:actin related protein 2/3 comp
GJB2:gap junction protein beta 2
SOCS2:suppressor of cytokine signali
SLC13A5:solute carrier family 13 membe
NFE2L2:nuclear factor, erythroid 2 li
IGFBP1:insulin like growth factor bin
HERPUD1:homocysteine inducible ER prot
GSR:glutathione−disulfide reductas
GREB1:growth regulating estrogen rec
GDF15:growth differentiation factor
CYP3A4:cytochrome P450 family 3 subfa
PECR:peroxisomal trans−2−enoyl−CoA
HPN:hepsin
TMEM185A:transmembrane protein 185A
SLC6A19:solute carrier family 6 member
LSS:lanosterol synthase
CAV1:caveolin 1
C1R:complement C1r
ACAT2:acetyl−CoA acetyltransferase 2
ABCC2:ATP binding cassette subfamily
TTR:transthyretin
TSKU:tsukushi, small leucine rich p
0
25
50
75
100
Number of Petroleum Substances (Out of 141)
Eliciting a Concentration Response
Direction
Down
Up

D

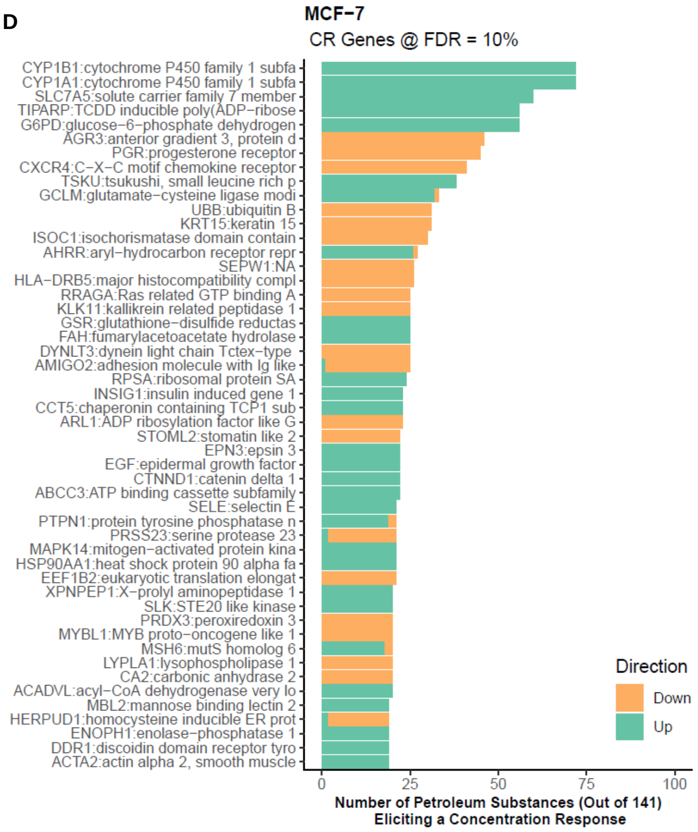
MCF−7
CR Genes @ FDR = 10%
CYP1B1:cytochrome P450 family 1 subfa
CYP1A1:cytochrome P450 family 1 subfa
SLC7A5:solute carrier family 7 member
TIPARP:TCDD inducible poly(ADP−ribose
G6PD:glucose−6−phosphate dehydrogen
AGR3:anterior gradient 3, protein d
PGR:progesterone receptor
CXCR4:C−X−C motif chemokine receptor
TSKU:tsukushi, small leucine rich p
GCLM:glutamate−cysteine ligase modi
UBB:ubiquitin B
KRT15:keratin 15
ISOC1:isochorismatase domain contain
AHRR:aryl−hydrocarbon receptor repr
SEPW1:NA
HLA−DRB5:major histocompatibility compl
RRAGA:Ras related GTP binding A
KLK11:kallikrein related peptidase 1
GSR:glutathione−disulfide reductas
FAH:fumarylacetoacetate hydrolase
DYNLT3:dynein light chain Tctex−type
AMIGO2:adhesion molecule with Ig like
RPSA:ribosomal protein SA
INSIG1:insulin induced gene 1
CCT5:chaperonin containing TCP1 sub
ARL1:ADP ribosylation factor like G
STOML2:stomatin like 2
EPN3:epsin 3
EGF:epidermal growth factor
CTNND1:catenin delta 1
ABCC3:ATP binding cassette subfamily
SELE:selectin E
PTPN1:protein tyrosine phosphatase n
PRSS23:serine protease 23
MAPK14:mitogen−activated protein kina
HSP90AA1:heat shock protein 90 alpha fa
EEF1B2:eukaryotic translation elongat
XPNPEP1:X−prolyl aminopeptidase 1
SLK:STE20 like kinase
PRDX3:peroxiredoxin 3
MYBL1:MYB proto−oncogene like 1
MSH6:mutS homolog 6
LYPLA1:lysophospholipase 1
CA2:carbonic anhydrase 2
ACADVL:acyl−CoA dehydrogenase very lo
MBL2:mannose binding lectin 2
HERPUD1:homocysteine inducible ER prot
ENOPH1:enolase−phosphatase 1
DDR1:discoidin domain receptor tyro
ACTA2:actin alpha 2, smooth muscle
0
25
50
75
100
Number of Petroleum Substances (Out of 141)
Eliciting a Concentration Response
Direction
Down
Up

E

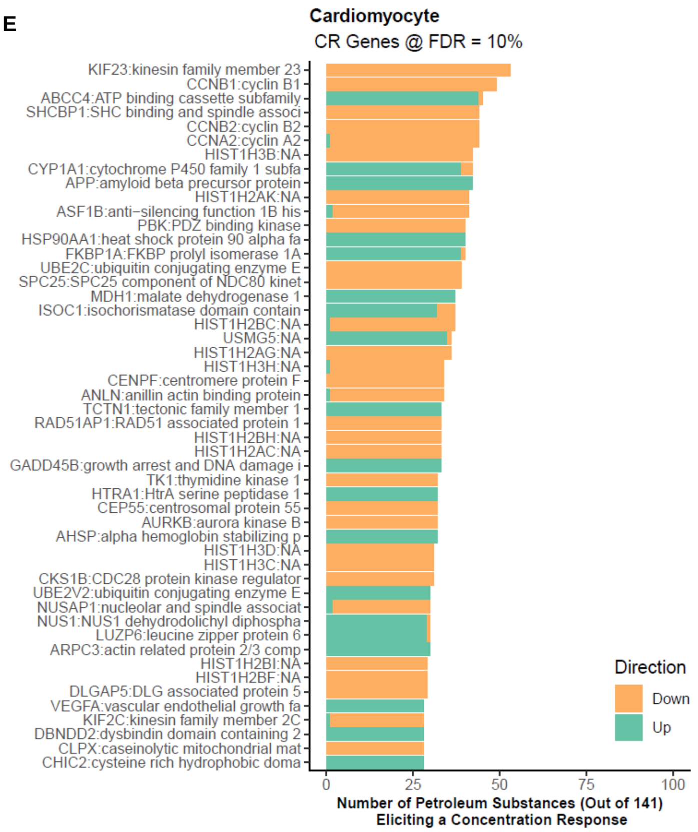
Cardiomyocyte
CR Genes @ FDR = 10%
KIF23:kinesin family member 23
CCNB1:cyclin B1
ABCC4:ATP binding cassette subfamily
SHCBP1:SHC binding and spindle associ
CCNB2:cyclin B2
CCNA2:cyclin A2
HIST1H3B:NA
CYP1A1:cytochrome P450 family 1 subfa
APP:amyloid beta precursor protein
HIST1H2AK:NA
ASF1B:anti−silencing function 1B his
PBK:PDZ binding kinase
HSP90AA1:heat shock protein 90 alpha fa
FKBP1A:FKBP prolyl isomerase 1A
UBE2C:ubiquitin conjugating enzyme E
SPC25:SPC25 component of NDC80 kinet
MDH1:malate dehydrogenase 1
ISOC1:isochorismatase domain contain
HIST1H2BC:NA
USMG5:NA
HIST1H2AG:NA
HIST1H3H:NA
CENPF:centromere protein F
ANLN:anillin actin binding protein
TCTN1:tectonic family member 1
RAD51AP1:RAD51 associated protein 1
HIST1H2BH:NA
HIST1H2AC:NA
GADD45B:growth arrest and DNA damage i
TK1:thymidine kinase 1
HTRA1:HtrA serine peptidase 1
CEP55:centrosomal protein 55
AURKB:aurora kinase B
AHSP:alpha hemoglobin stabilizing p
HIST1H3D:NA
HIST1H3C:NA
CKS1B:CDC28 protein kinase regulator
UBE2V2:ubiquitin conjugating enzyme E
NUSAP1:nucleolar and spindle associat
NUS1:NUS1 dehydrodolichyl diphospha
LUZP6:leucine zipper protein 6
ARPC3:actin related protein 2/3 comp
HIST1H2BI:NA
HIST1H2BF:NA
DLGAP5:DLG associated protein 5
VEGFA:vascular endothelial growth fa
KIF2C:kinesin family member 2C
DBNDD2:dysbindin domain containing 2
CLPX:caseinolytic mitochondrial mat
CHIC2:cysteine rich hydrophobic doma
0
25
50
75
100
Number of Petroleum Substances (Out of 141)
Eliciting a Concentration Response
Direction
Down
Up

F

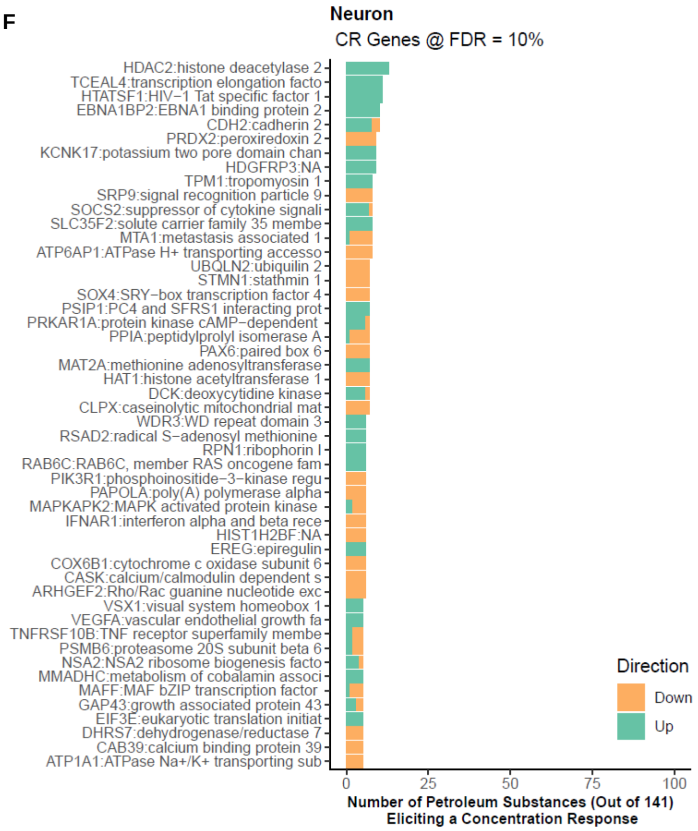
Neuron
CR Genes @ FDR = 10%
HDAC2:histone deacetylase 2
TCEAL4:transcription elongation facto
HTATSF1:HIV−1 Tat specific factor 1
EBNA1BP2:EBNA1 binding protein 2
CDH2:cadherin 2
PRDX2:peroxiredoxin 2
KCNK17:potassium two pore domain chan
HDGFRP3:NA
TPM1:tropomyosin 1
SRP9:signal recognition particle 9
SOCS2:suppressor of cytokine signali
SLC35F2:solute carrier family 35 membe
MTA1:metastasis associated 1
ATP6AP1:ATPase H+ transporting accesso
UBQLN2:ubiquilin 2
STMN1:stathmin 1
SOX4:SRY−box transcription factor 4
PSIP1:PC4 and SFRS1 interacting prot
PRKAR1A:protein kinase cAMP−dependent
PPIA:peptidylprolyl isomerase A
PAX6:paired box 6
MAT2A:methionine adenosyltransferase
HAT1:histone acetyltransferase 1
DCK:deoxycytidine kinase
CLPX:caseinolytic mitochondrial mat
WDR3:WD repeat domain 3
RSAD2:radical S−adenosyl methionine
RPN1:ribophorin I
RAB6C:RAB6C, member RAS oncogene fam
PIK3R1:phosphoinositide−3−kinase regu
PAPOLA:poly(A) polymerase alpha
MAPKAPK2:MAPK activated protein kinase
IFNAR1:interferon alpha and beta rece
HIST1H2BF:NA
EREG:epiregulin
COX6B1:cytochrome c oxidase subunit 6
CASK:calcium/calmodulin dependent s
ARHGEF2:Rho/Rac guanine nucleotide exc
VSX1:visual system homeobox 1
VEGFA:vascular endothelial growth fa
TNFRSF10B:TNF receptor superfamily membe
PSMB6:proteasome 20S subunit beta 6
NSA2:NSA2 ribosome biogenesis facto
MMADHC:metabolism of cobalamin associ
MAFF:MAF bZIP transcription factor
GAP43:growth associated protein 43
EIF3E:eukaryotic translation initiat
DHRS7:dehydrogenase/reductase 7
CAB39:calcium binding protein 39
ATP1A1:ATPase Na+/K+ transporting sub
0
25
50
75
100
Number of Petroleum Substances (Out of 141)
Eliciting a Concentration Response
Direction
Down
Up

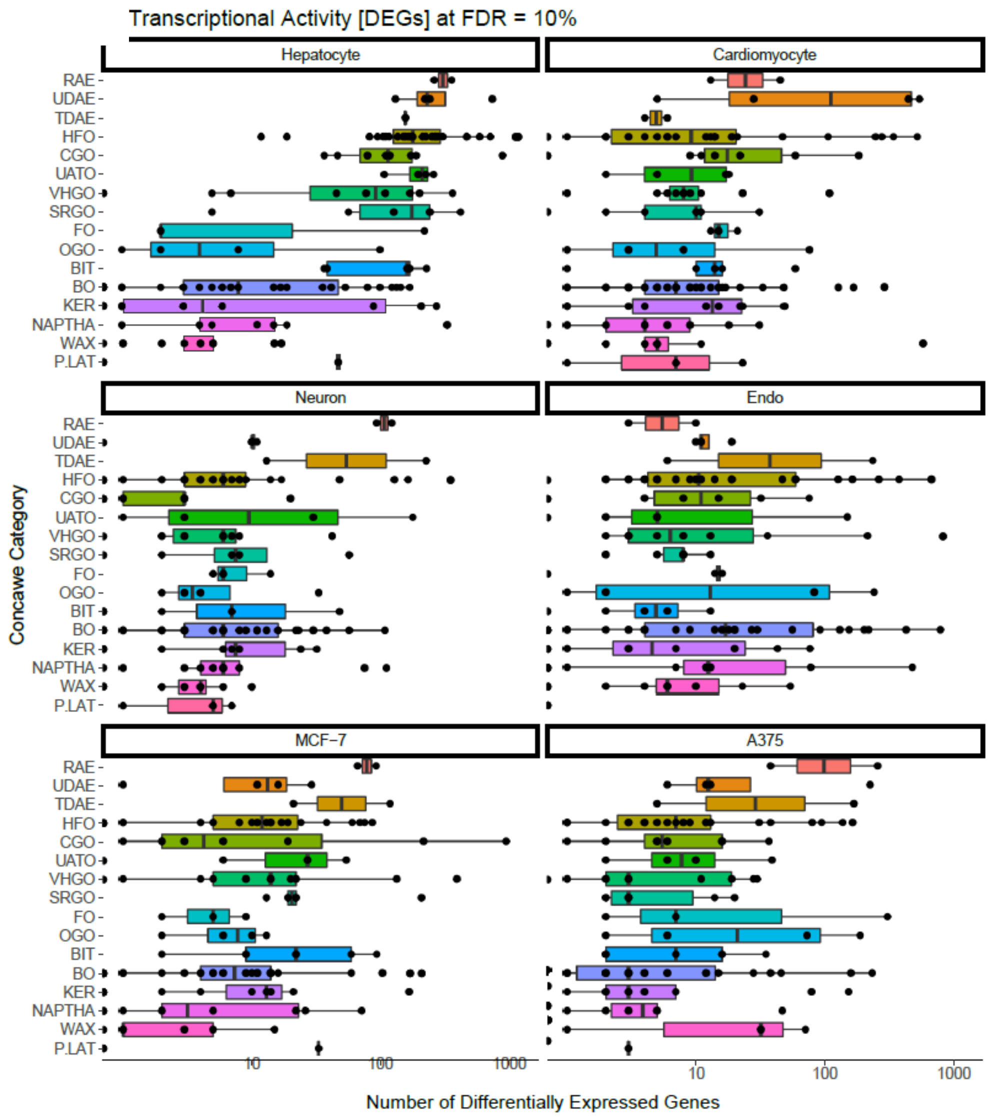

Fig. S3: Class-specific effects of petroleum substances on gene expression in the multi-cell *in vitro* transcriptomic analysis by cell type

Box and whiskers plots show the range in the number of genes significantly (FDR ≤ 10%) differentially expressed by the substances in each class when comparing maximum dose to method blank controls.

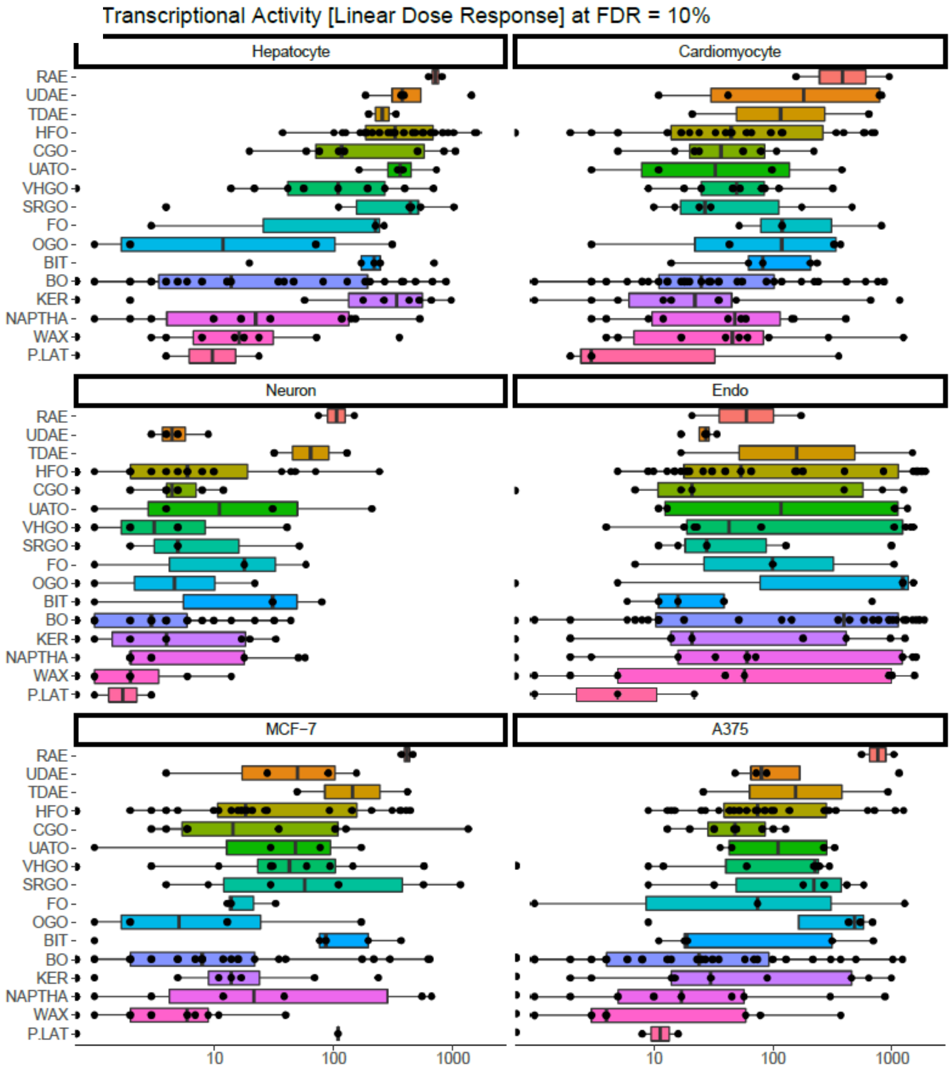

Fig. S4: Class-specific effects of petroleum substances on gene expression throughout a concentration-response in the multi-cell *in vitro* transcriptomic analysis by celltype

Box and whiskers plots show the range in the number of genes significantly (FDR≤10%) exhibiting a concentration response by the substances in each class when comparing maximum dose to method blank controls.

A

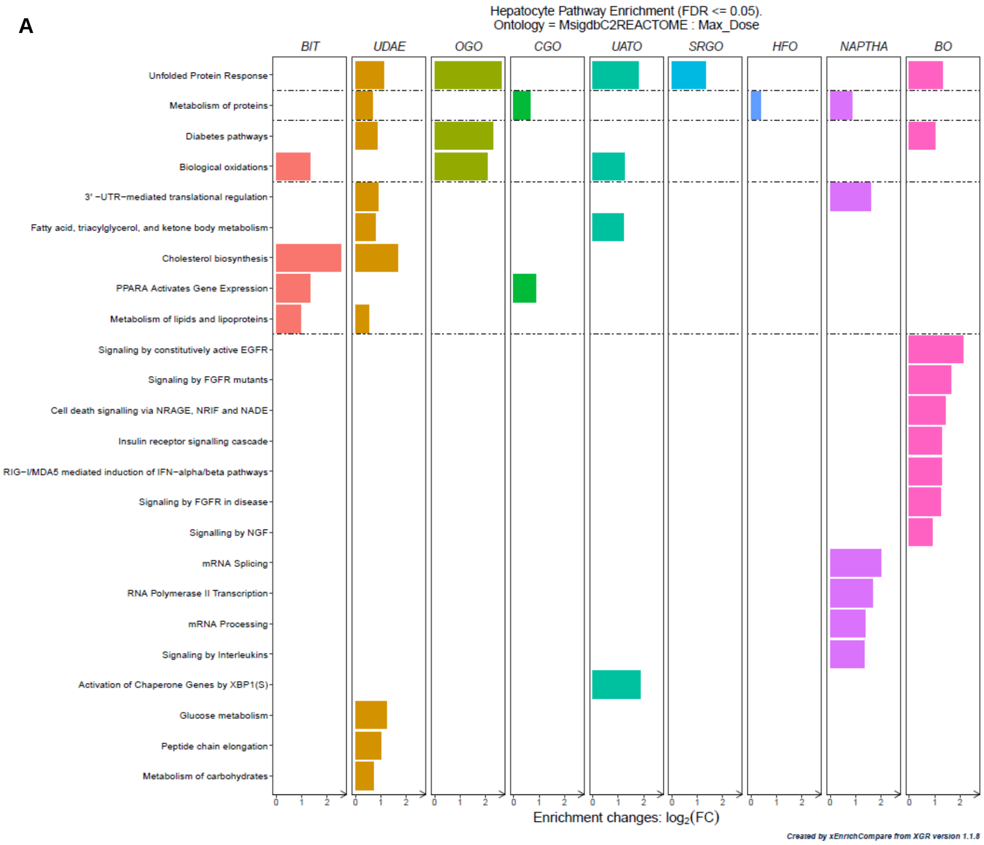

B

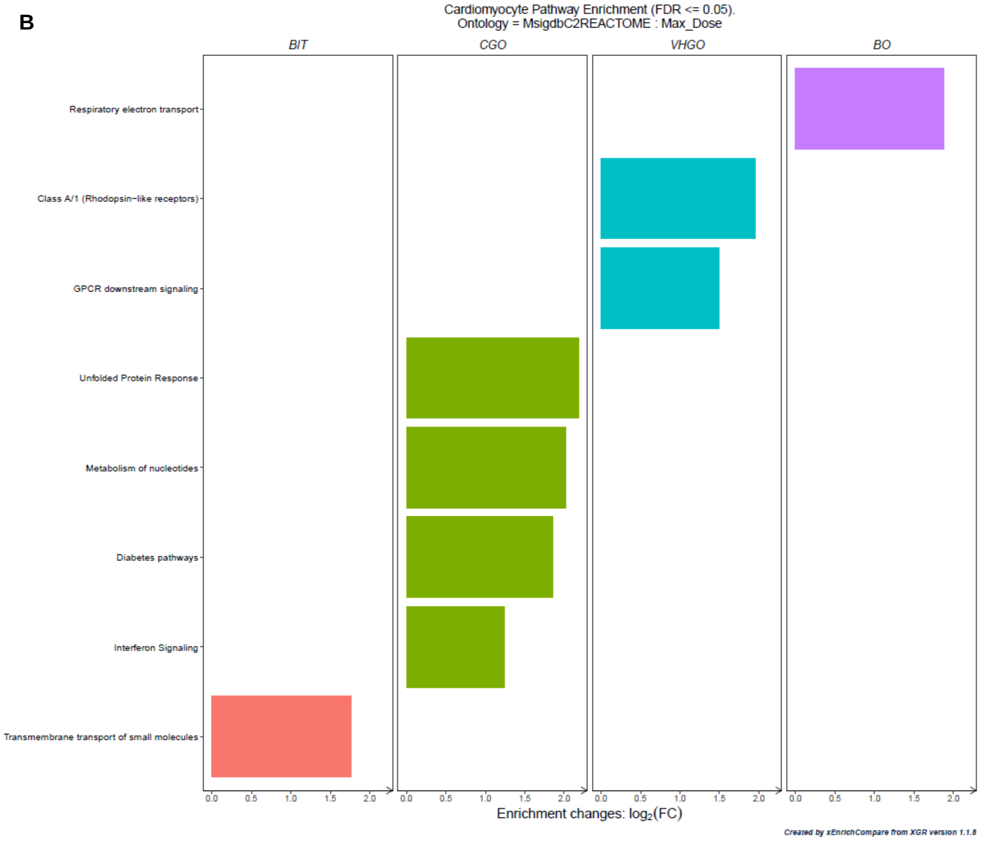

Fig. S5: Class-specific effects of petroleum substances on pathway enrichment in the multi-cell *in vitro* transcriptomic analysis and differential gene expression at max. dose

An FDR of 5% was used to select any genes differentially expressed for any substance within a category. Pathway analysis using C2Reactome ontologies was conducted with an FDR of 5% applied for pathway analysis. Bar plots show enriched C2Reactome ontologies by cell type. The background was set to all genes assessed after QC by each cell type.

C

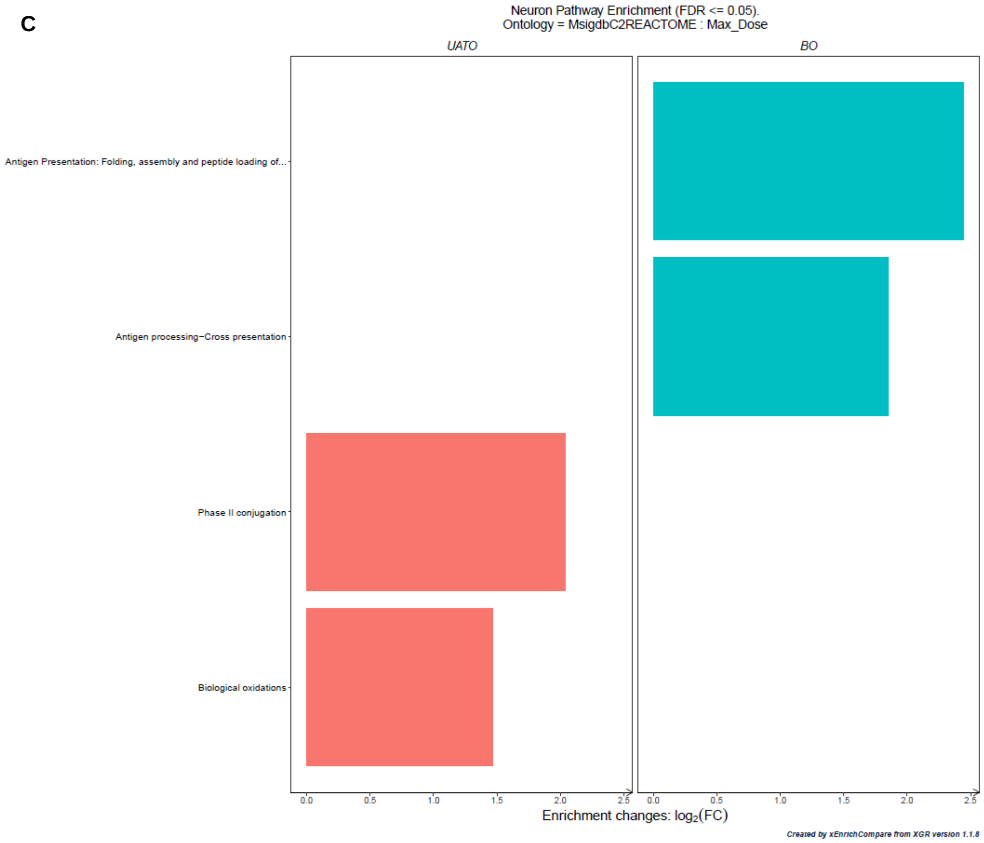

Neuron Pathway Enrichment (FDR <= 0.05).
Ontology = MsigdbC2REACTOME : Max_Dose
UATO
BO
Antigen Presentation: Folding, assembly and peptide loading of...
Antigen processing−Cross presentation
Phase II conjugation
Biological oxidations
0.0
0.5
1.0
1.5
2.0
2.5
Enrichment changes: log2(FC)
Created by xEnrichCompare from XGR version 1.1.8

D

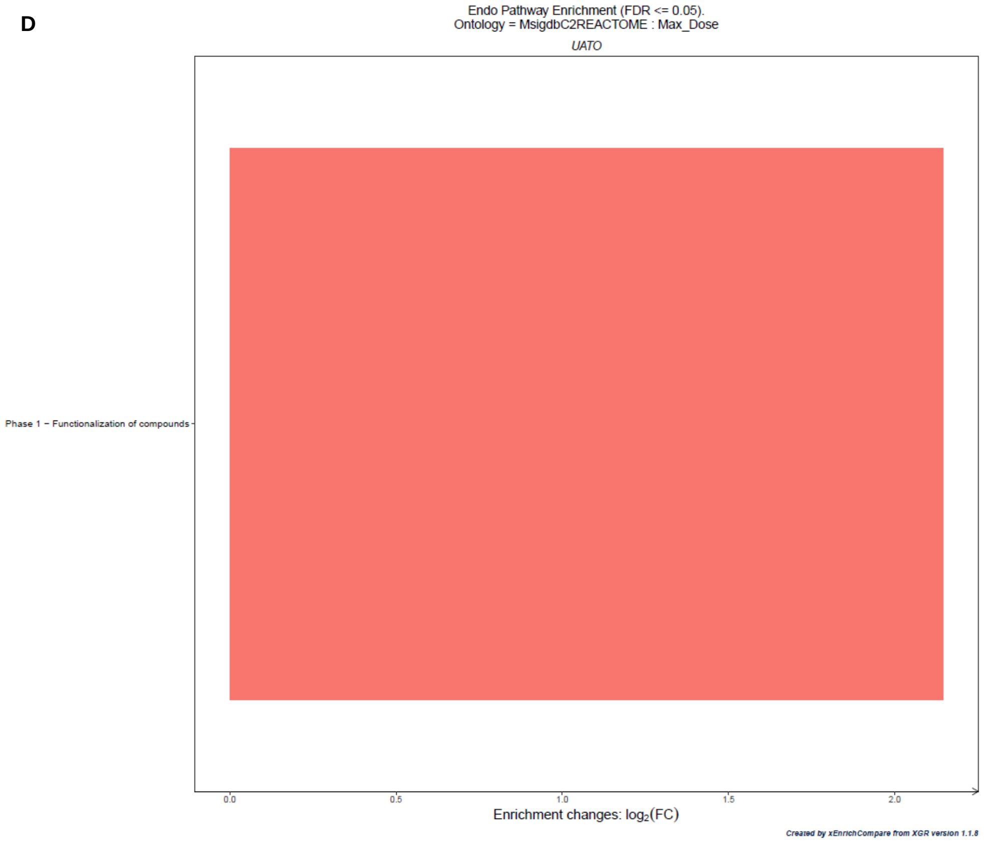

Endo Pathway Enrichment (FDR <= 0.05).
Ontology = MsigdbC2REACTOME : Max_Dose
UATO
Phase 1 − Functionalization of compounds
0.0
0.5
1.0
1.5
2.0
Enrichment changes: log2(FC)
Created by xEnrichCompare from XGR version 1.1.8

E

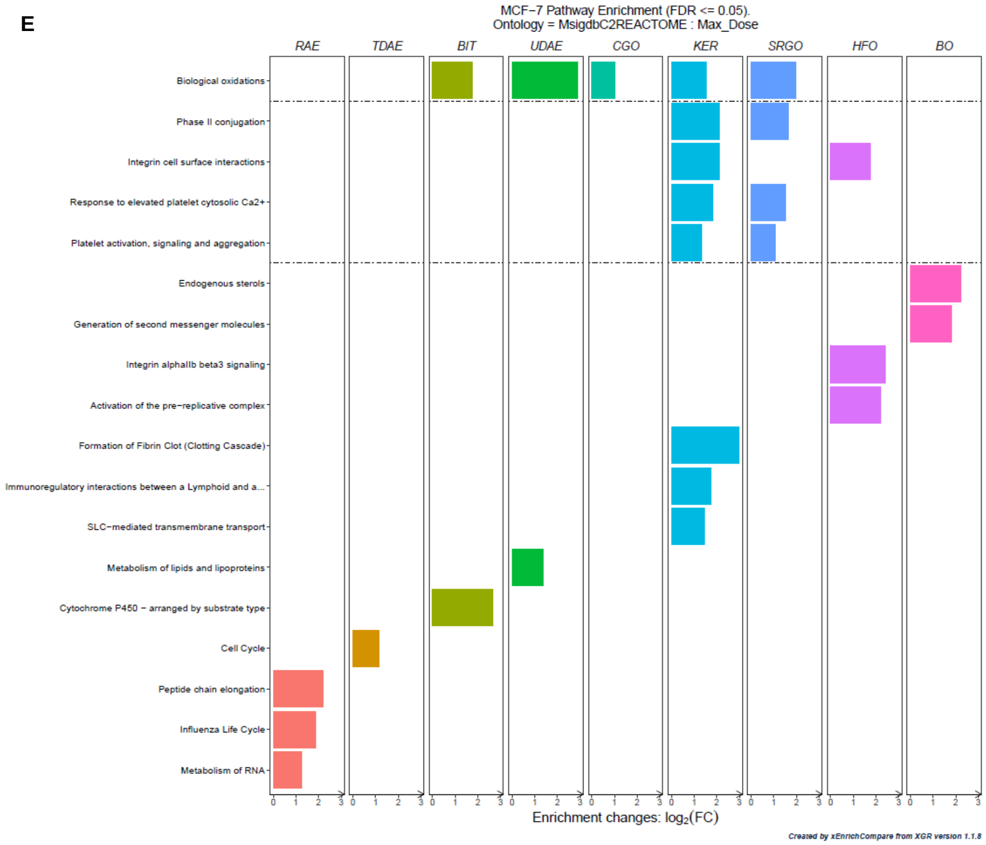

MCF−7 Pathway Enrichment (FDR <= 0.05).
Ontology = MsigdbC2REACTOME : Max_Dose
RAE
TDAE
BIT
UDAE
CGO
KER
SRGO
HFO
BO
Biological oxidations
Phase II conjugation
Integrin cell surface interactions
Response to elevated platelet cytosolic Ca2+
Platelet activation, signaling and aggregation
Endogenous sterols
Generation of second messenger molecules
Integrin alphaIIb beta3 signaling
Activation of the pre−replicative complex
Formation of Fibrin Clot (Clotting Cascade)
Immunoregulatory interactions between a Lymphoid and a...
SLC−mediated transmembrane transport
Metabolism of lipids and lipoproteins
Cytochrome P450 − arranged by substrate type
Cell Cycle
Peptide chain elongation
Influenza Life Cycle
Metabolism of RNA
Enrichment changes: log2(FC)
Created by xEnrichCompare from XGR version 1.1.8

F

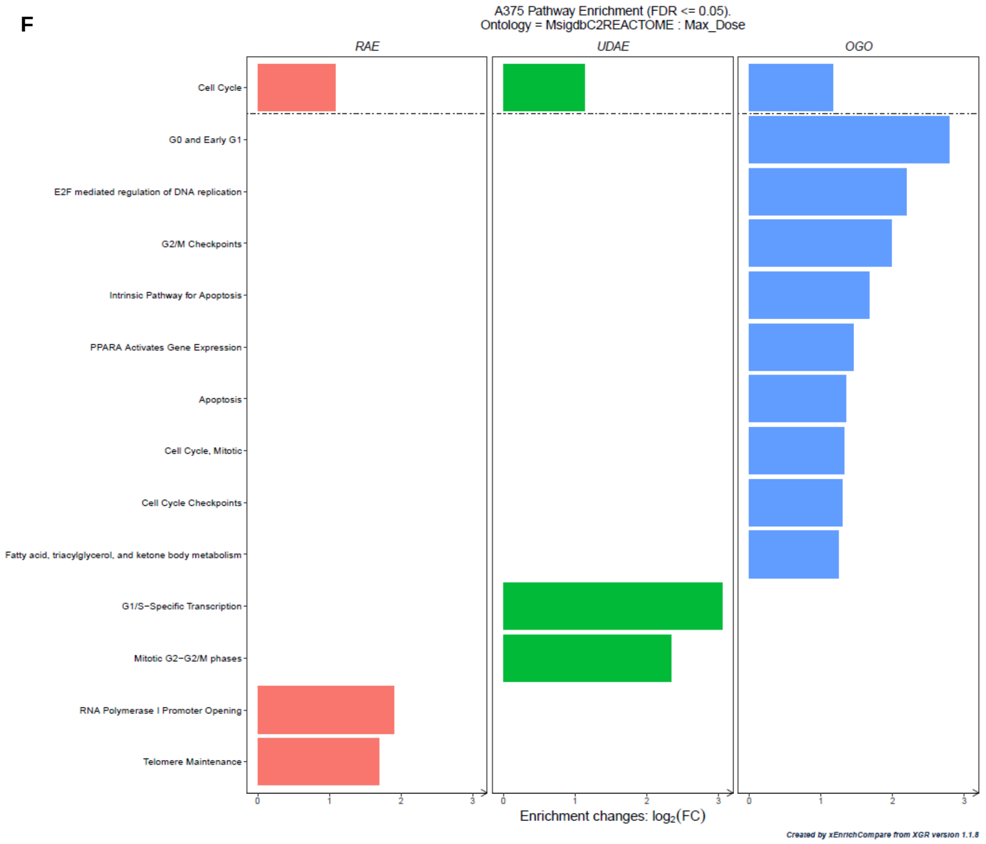

A375 Pathway Enrichment (FDR <= 0.05).
Ontology = MsigdbC2REACTOME : Max_Dose
RAE
UDAE
OGO
Cell Cycle
G0 and Early G1
E2F mediated regulation of DNA replication
G2/M Checkpoints
Intrinsic Pathway for Apoptosis
PPARA Activates Gene Expression
Apoptosis
Cell Cycle, Mitotic
Cell Cycle Checkpoints
Fatty acid, triacylglycerol, and ketone body metabolism
G1/S−Specific Transcription
Mitotic G2−G2/M phases
RNA Polymerase I Promoter Opening
Telomere Maintenance
Enrichment changes: log2(FC)
Created by xEnrichCompare from XGR version 1.1.8

A

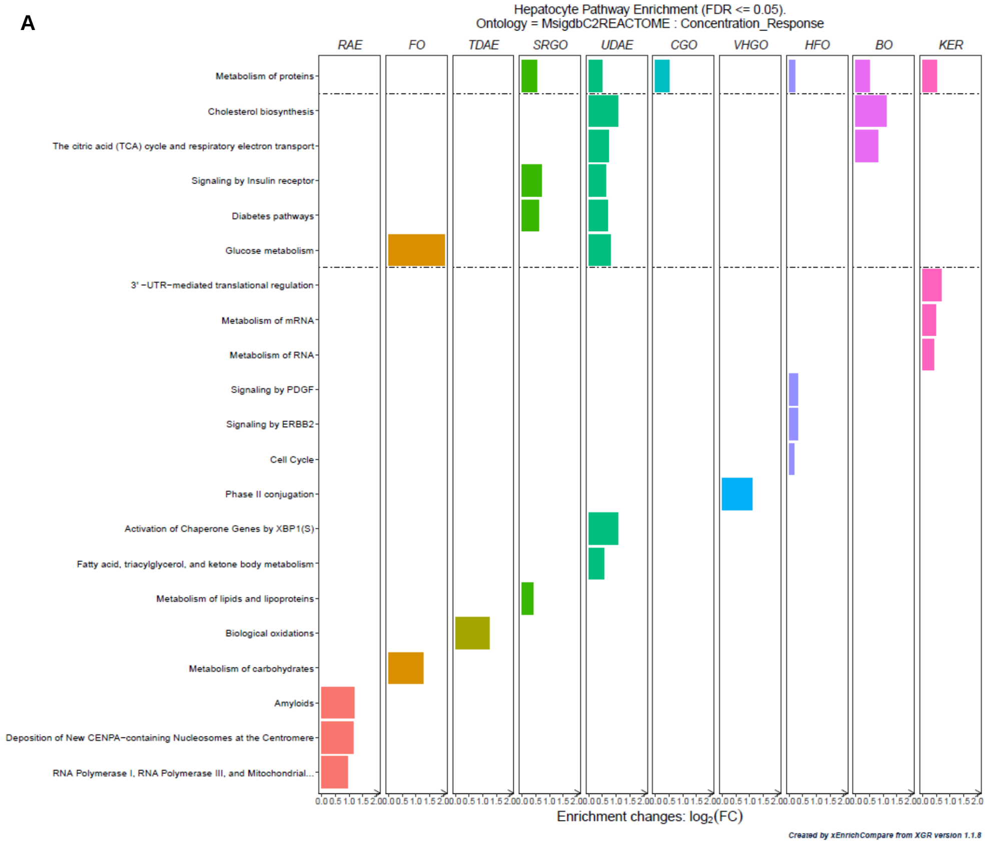

B

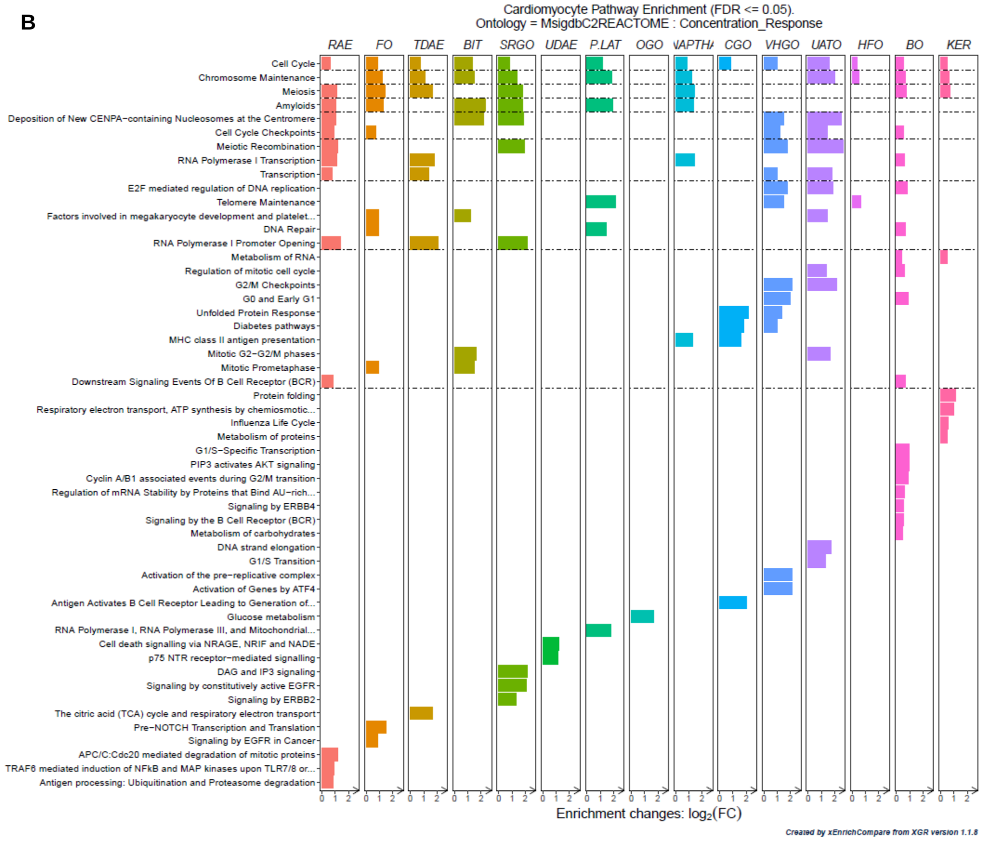

Fig. S6: Class-specific effects of petroleum substances on pathway enrichment in the multi-cell *in vitro* transcriptomic analysis of concentration response
An FDR of 5% was used to select any concentration-responsive genes for any substance within a category. Pathway analysis using C2Reactome ontologies was conducted with an FDR of 5% applied for pathway analysis. Bar plots show enriched C2Reactome ontologies by cell type. The background was set to all genes assessed after QC by each cell type.

C

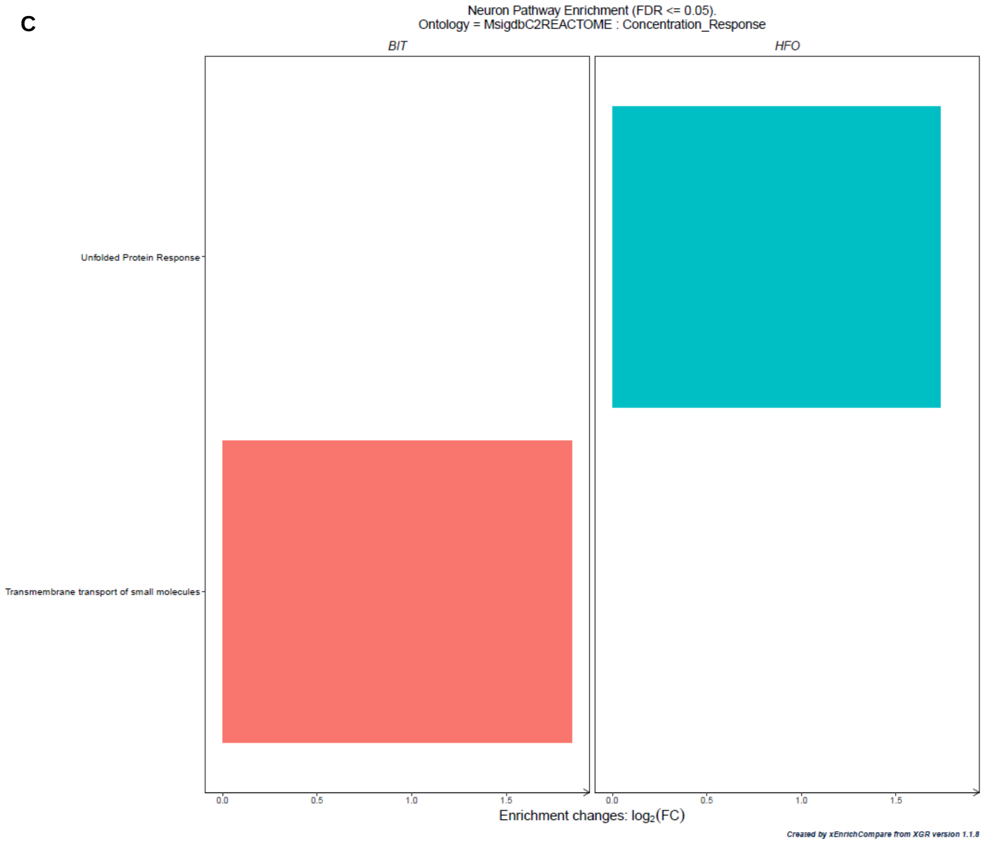
Neuron Pathway Enrichment (FDR <= 0.05).
Ontology = MsigdbC2REACTOME : Concentration_Response
BIT
HFO
Unfolded Protein Response
Transmembrane transport of small molecules
0.0
0.5
1.0
1.5
Enrichment changes: log2(FC)
Created by xEnrichCompare from XGR version 1.1.8

D

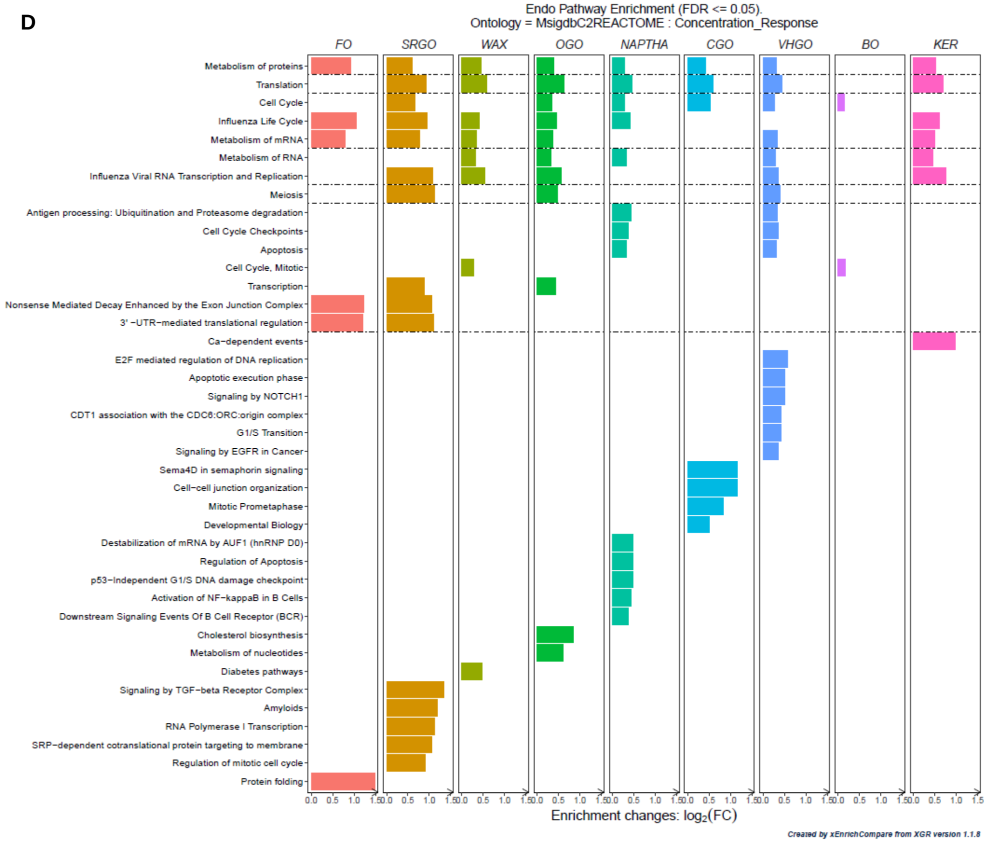
Endo Pathway Enrichment (FDR <= 0.05).
Ontology = MsigdbC2REACTOME : Concentration_Response
FO
SRGO
WAX
OGO
NAPTHA
CGO
VHGO
BO
KER
Metabolism of proteins
Translation
Cell Cycle
Influenza Life Cycle
Metabolism of mRNA
Metabolism of RNA
Influenza Viral RNA Transcription and Replication
Meiosis
Antigen processing: Ubiquitination and Proteasome degradation
Cell Cycle Checkpoints
Apoptosis
Cell Cycle, Mitotic
Transcription
Nonsense Mediated Decay Enhanced by the Exon Junction Complex
3' −UTR−mediated translational regulation
Ca−dependent events
E2F mediated regulation of DNA replication
Apoptotic execution phase
Signaling by NOTCH1
CDT1 association with the CDC6:ORC:origin complex
G1/S Transition
Signaling by EGFR in Cancer
Sema4D in semaphorin signaling
Cell−cell junction organization
Mitotic Prometaphase
Developmental Biology
Destabilization of mRNA by AUF1 (hnRNP D0)
Regulation of Apoptosis
p53−Independent G1/S DNA damage checkpoint
Activation of NF−kappaB in B Cells
Downstream Signaling Events Of B Cell Receptor (BCR)
Cholesterol biosynthesis
Metabolism of nucleotides
Diabetes pathways
Signaling by TGF−beta Receptor Complex
Amyloids
RNA Polymerase I Transcription
SRP−dependent cotranslational protein targeting to membrane
Regulation of mitotic cell cycle
Protein folding
0.0
0.5
1.0
1.5
Enrichment changes: log2(FC)
Created by xEnrichCompare from XGR version 1.1.8

E

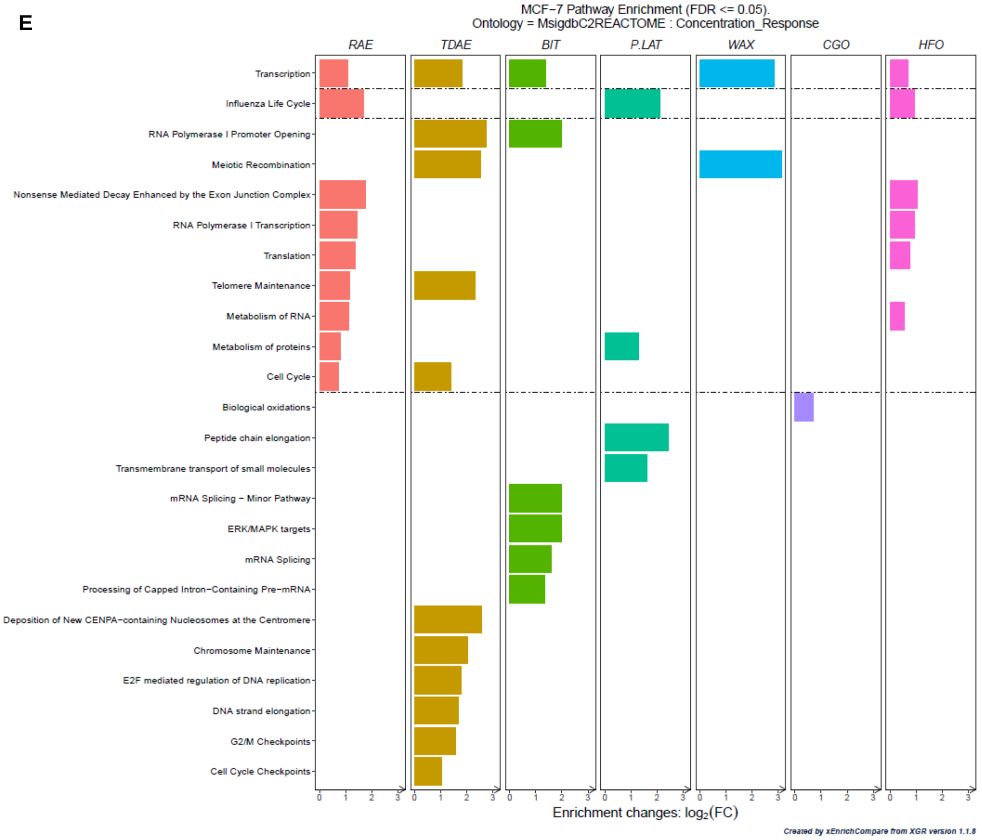
MCF−7 Pathway Enrichment (FDR <= 0.05).
Ontology = MsigdbC2REACTOME : Concentration_Response
RAE
TDAE
BIT
P.LAT
WAX
CGO
HFO
Transcription
Influenza Life Cycle
RNA Polymerase I Promoter Opening
Meiotic Recombination
Nonsense Mediated Decay Enhanced by the Exon Junction Complex
RNA Polymerase I Transcription
Translation
Telomere Maintenance
Metabolism of RNA
Metabolism of proteins
Cell Cycle
Biological oxidations
Peptide chain elongation
Transmembrane transport of small molecules
mRNA Splicing − Minor Pathway
ERK/MAPK targets
mRNA Splicing
Processing of Capped Intron−Containing Pre−mRNA
Deposition of New CENPA−containing Nucleosomes at the Centromere
Chromosome Maintenance
E2F mediated regulation of DNA replication
DNA strand elongation
G2/M Checkpoints
Cell Cycle Checkpoints
Enrichment changes: log2(FC)
Created by xEnrichCompare from XGR version 1.1.8

F

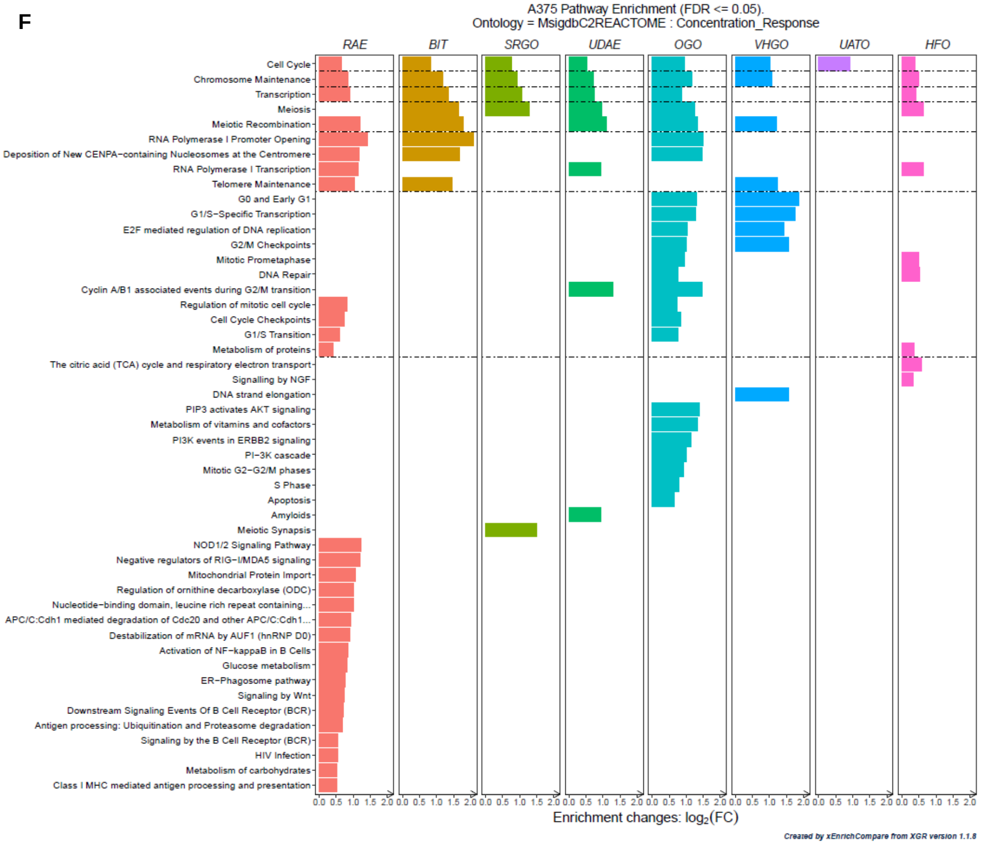
A375 Pathway Enrichment (FDR <= 0.05).
Ontology = MsigdbC2REACTOME : Concentration_Response
RAE
BIT
SRGO
UDAE
OGO
VHGO
UATO
HFO
Cell Cycle
Chromosome Maintenance
Transcription
Meiosis
Meiotic Recombination
RNA Polymerase I Promoter Opening
Deposition of New CENPA−containing Nucleosomes at the Centromere
RNA Polymerase I Transcription
Telomere Maintenance
G0 and Early G1
G1/S−Specific Transcription
E2F mediated regulation of DNA replication
G2/M Checkpoints
Mitotic Prometaphase
DNA Repair
Cyclin A/B1 associated events during G2/M transition
Regulation of mitotic cell cycle
Cell Cycle Checkpoints
G1/S Transition
Metabolism of proteins
The citric acid (TCA) cycle and respiratory electron transport
Signalling by NGF
DNA strand elongation
PIP3 activates AKT signaling
Metabolism of vitamins and cofactors
PI3K events in ERBB2 signaling
PI−3K cascade
Mitotic G2−G2/M phases
S Phase
Apoptosis
Amyloids
Meiotic Synapsis
NOD1/2 Signaling Pathway
Negative regulators of RIG−I/MDA5 signaling
Mitochondrial Protein Import
Regulation of ornithine decarboxylase (ODC)
Nucleotide−binding domain, leucine rich repeat containing...
APC/C:Cdh1 mediated degradation of Cdc20 and other APC/C:Cdh1...
Destabilization of mRNA by AUF1 (hnRNP D0)
Activation of NF−kappaB in B Cells
Glucose metabolism
ER−Phagosome pathway
Signaling by Wnt
Downstream Signaling Events Of B Cell Receptor (BCR)
Antigen processing: Ubiquitination and Proteasome degradation
Signaling by the B Cell Receptor (BCR)
HIV Infection
Metabolism of carbohydrates
Class I MHC mediated antigen processing and presentation
Enrichment changes: log2(FC)
Created by xEnrichCompare from XGR version 1.1.8
